# Comparative analysis of the alveolar microbiome in COPD, ECOPD, Sarcoidosis, and ILD patients to identify respiratory illnesses specific microbial signatures

**DOI:** 10.1038/s41598-021-83524-2

**Published:** 2021-02-17

**Authors:** Shashank Gupta, Malini Shariff, Gaura Chaturvedi, Agrima Sharma, Nitin Goel, Monika Yadav, Martin S. Mortensen, Søren J. Sørensen, Mitali Mukerji, Nar Singh Chauhan

**Affiliations:** 1 Section of Microbiology, Department of Biology, University of Copenhagen, 2100 Copenhagen, Denmark; 2 Department of Microbiology, Vallabhbhai Patel Chest Institute, University of Delhi, Delhi, 110007 India; 3 Genomics and Molecular Medicine, CSIR-Institute of Genomics and Integrative Biology (CSIR -IGIB), South Campus, Sukhdev Vihar, Mathura Road, New Delhi, 110025 India; 4 Academy of Scientific and Innovative Research (AcSIR), Ghaziabad, 201002 India; 5 Department of Pulmonary Medicine, Vallabhbhai Patel Chest Institute, University of Delhi, Delhi, 110007 India; 6 Department of Biochemistry, Maharshi Dayanand University, Rohtak, Haryana 124001 India

**Keywords:** Metagenomics, Microbiome, Microbial communities, Symbiosis

## Abstract

Studying respiratory illness-specific microbial signatures and their interaction with other micro-residents could provide a better understanding of lung microbial ecology. Each respiratory illness has a specific disease etiology, however, so far no study has revealed disease—specific microbial markers. The present study was designed to determine disease-specific microbial features and their interactions with other residents in chronic obstructive pulmonary diseases (stable and exacerbated), sarcoidosis, and interstitial lung diseases. Broncho-alveolar lavage samples (n = 43) were analyzed by SSU rRNA gene sequencing to study the alveolar microbiome in these diseases. A predominance of Proteobacteria followed by Firmicutes, Bacteroidetes, Actinobacteria, and Fusobacteria was observed in all the disease subsets. Shannon diversity was significantly higher in stable COPD when compared to exacerbated chronic obstructive pulmonary disease (ECOPD) (*p* = 0.0061), and ILD patient samples (*p* = 0.037). The lung microbiome of the patients with stable COPD was more diverse in comparison to ECOPD and ILD patients (*p* < 0.001). Lefse analysis identified 40 disease—differentiating microbial features (LDA score (log10) > 4). Species network analysis indicated a significant correlation (*p* < 0.05) of diseases specific microbial signature with other lung microbiome members. The current study strengthens the proposed hypothesis that each respiratory illness has unique microbial signatures. These microbial signatures could be used as diagnostic markers to differentiate among various respiratory illnesses.

## Introduction

Chronic obstructive pulmonary disease (COPD), interstitial lung diseases (ILD), sarcoidosis are dynamic, debilitating lung diseases with multiple comorbidities that affect millions of people worldwide^1–3^. COPD is characterized by persistent respiratory symptoms and airflow limitations due to airway and/or alveolar abnormalities^4^. Infections can further weaken the airway function and lead to the exacerbations of COPD^5^. ILD is a heterogeneous group of respiratory disorders presenting with dyspnea, cough, and/or impaired pulmonary function^6^. Radiologic and histopathologic evaluation of the lungs shows patterns of inflammation and fibrosis among ILD patients^7,8^.

These pathophysiological disorders alter lung physiology and could induce lung microbial dysbiosis^9^. Studies have been initiated to define lung microbiome composition in health and disease subsets to identify microbial markers for disease prognosis and timely therapeutic interventions^10–13^. Assessment of the temporal and spatial organization of lung microbes^14^ and the disease-associated key microbes have also been reported^15,16^. Several studies have indicated lung microbial dysbiosis during the onset of various pathophysiological diseases when compared to healthy controls^11,14–22^. For instance, an abundance of *Streptococcus*, *Corynebacterium*, *Alloiococcus*, *Prevotella, Veillonella*, *Rothia*, *Porphyromonas*, and *Moraxella* were associated with COPD patients^14,15,17^. *Haemophilus, Pseudomonas*, and *Moraxella* microbial groups are reported to be enriched in the lung microbiome during the onset of exacerbated COPD^14,15,17,18^. Similarly, an abundance of *Veillonella*, *Megasphaera*, *Streptococcus*, *Prevotella*, *Acidovorax* was observed in the lung microbiome of patients with lung cancer^19^. Lung microbiome of the asthma patients showed enrichment of *Haemophilus*, *Moraxella*, *Neisseria*, *Streptococcus,* and *Staphylococcus* microbial species^20^. *Streptococcus*, *Prevotella*, *Veillonella*, *Rothia*, *Actinomyces*, *Gemella*, *Granulicatella*, *Fusobacterium*, *Neisseria*, and *Atopobium* species are abundant in the lung microbiome of the Cystic fibrosis patients^21^. The lung microbiome of the sarcoidosis patients has the enrichment of *Atopobium* and *Fusobacterium* species^22^.

These studies have indicated lung microbial dysbiosis during the onset of various pathophysiological disorders. Despite varied etiology, different pathophysiological conditions showed enrichment of almost similar microbial groups in each disease subset. *Streptococcus*, *Prevotella, Veillonella*, *Rothia*, and *Moraxella* are over-represented in the lung microbiome of patients with COPD, cystic fibrosis, asthma, and lung cancer^14–22^. Similarly, *Atopobium* and *Fusobacterium* are found enriched within the lung microbiome of patients with cystic fibrosis, sarcoidosis, and ILD^21,22^. These overlapping results limit the applicability of this information to develop respiratory illness-specific molecular diagnostics. We hypothesized that patients with stable COPD, ECOPD (exacerbated COPD), sarcoidosis, and other ILDs have varied disease etiology and each disease could have a unique lung microbiome profile. The current study was designed to explore the composition and distribution of microbial phylotypes in the disturbed physiological states of the lungs. A comparative lung microbiome analysis between diseases, instead of comparison with healthy individuals could help to identify respiratory illness-specific microbial markers that can be used for diseases-specific diagnosis. This attempt is a first of its kind to conduct an alveolar lung microbiome comparison among disease subsets.

## Results

### Quality of the sequencing dataset

Fourteen patients with stable COPD, thirteen patients with exacerbations of COPD, eight patients with ILD, and eight patients with sarcoidosis were enrolled in this study (Table 1, Supplementary Tables S1, S2, S3). A total of 1,282,459 raw reads were passed through the quality filter and chimera detection resulting in 772,133 (mean per sample: 17,956 ± 1651) high quality and non-chimeric reads. Based on dada2, amplicons were clustered into 2329 amplicon sequence variants (ASVs). The coverage of our sequencing was assessed by rarefaction curves (Supplementary Fig. S1). ASV tables were rarefied to 4,351 reads per sample to remove the sequencing biases and represent 2162 ASVs across the 43 samples.

**Table 1:** Characteristics of the participants in this study.

	ECOPD (n = 13)	Stable COPD (n = 14)	Sarcoidosis (n = 8)	ILD (n = 8)
Age, years	63.6 ± 5.79	53.6 ± 14.3	44.5 ± 13.1	53.5 ± 10.9
Sex (% male)	76.9	100	62.5	37.5
Smoker (n)	8	10 (3NA*)	1 (1NA*)	2
BME	5 (2NA*)	0 (3NA*)	3 (1NA*)	2

Data are presented as percentage value or mean ± SD as appropriate.

BME: Biomass Exposure.

*Data not available.

### Alveolar microbiome composition

**Figure 1:**
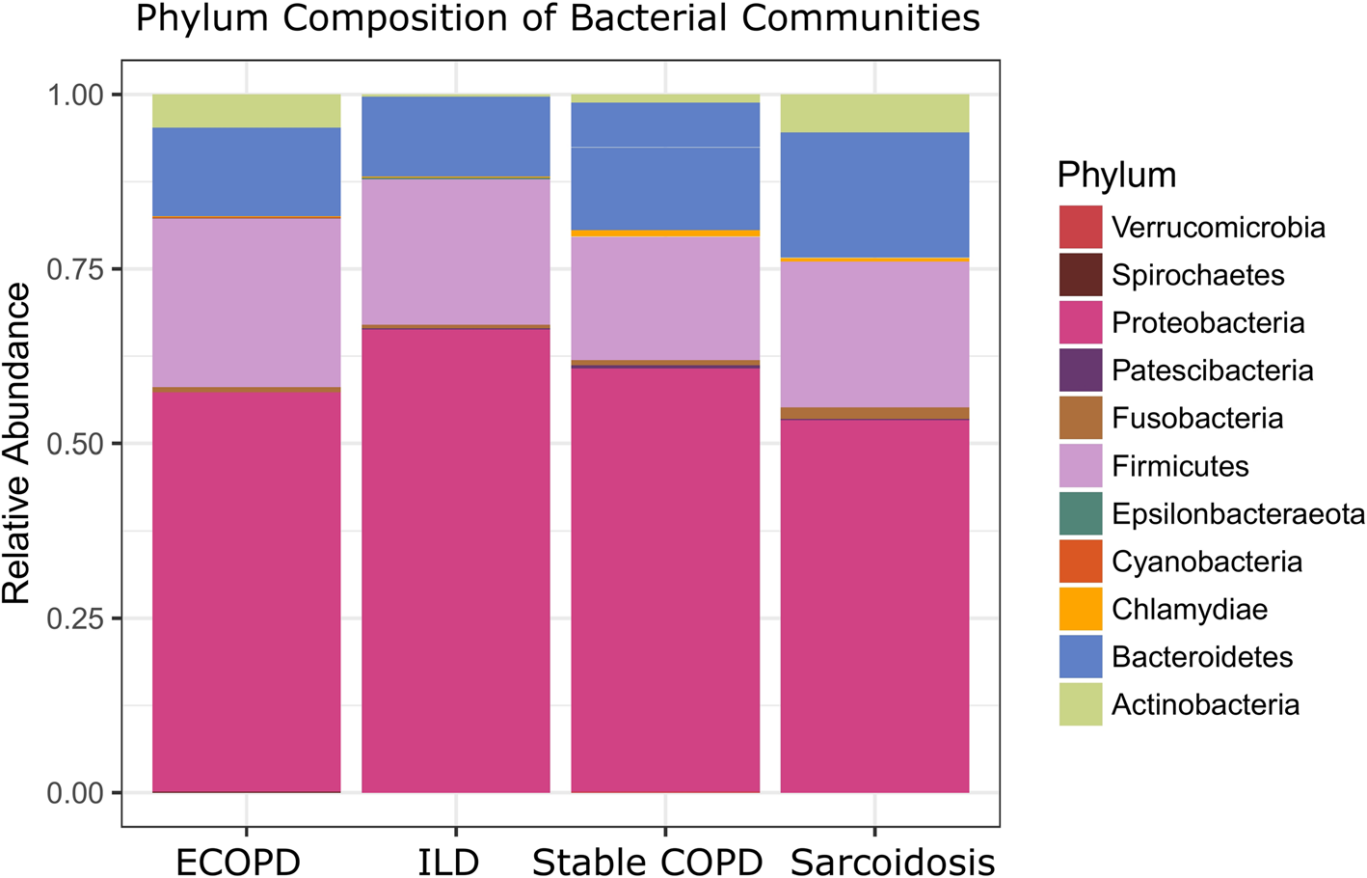
Microbiota composition at phylum level in each disease group. Stacked bar plot showing the mean relative abundance, at phylum level, for each disease. Phyla with a mean relative abundance below 1% for all diseases were excluded from the plot.

**Figure 2:**
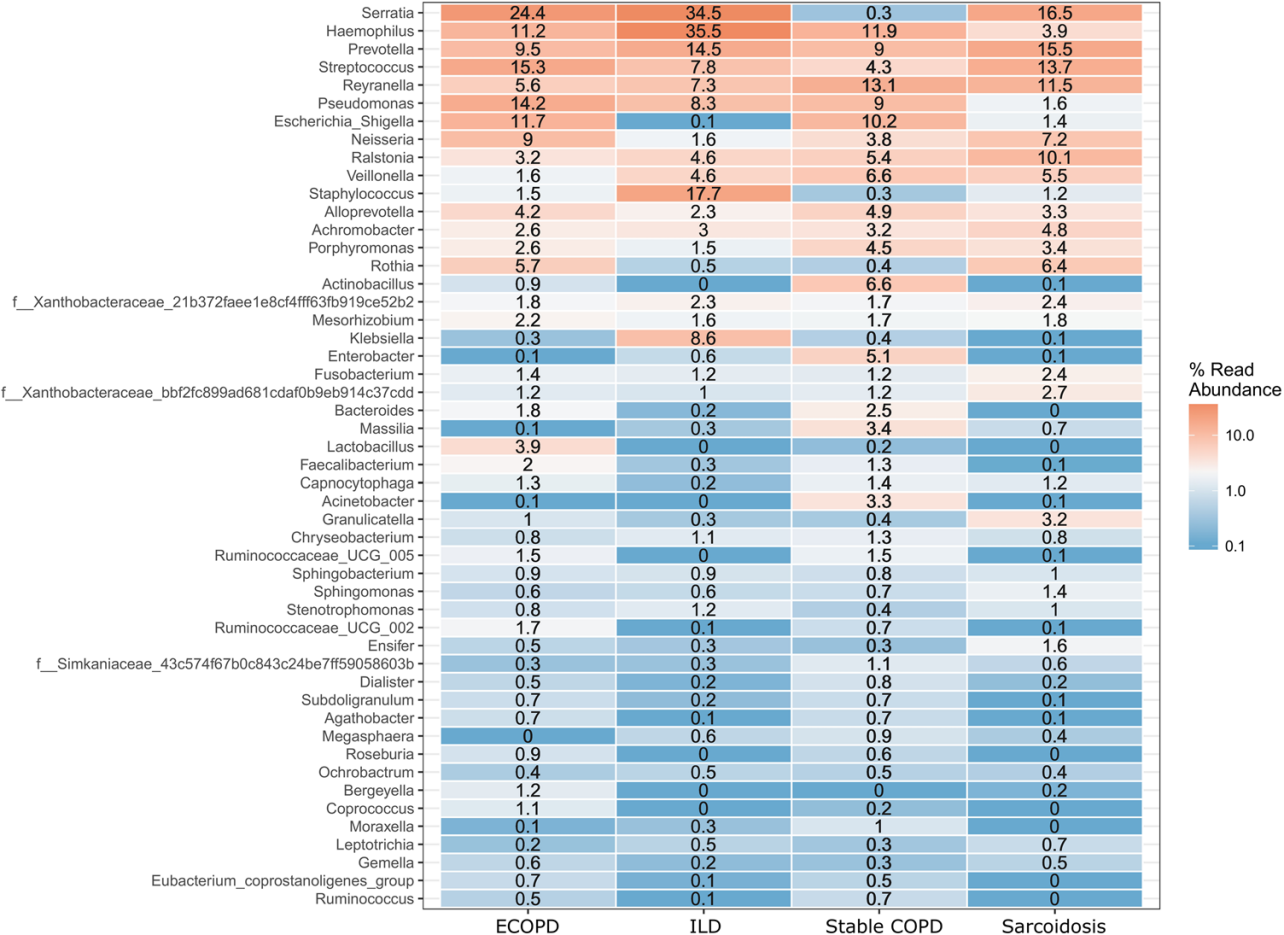
Heat map showing 50 most abundant genera in the four groups of samples. Columns represent the groups and rows the genera and their relative abundance. The color key represents the relative abundance of each genus.

Microbial diversity analysis among different disease groups identified the prevalence of 19 bacterial phyla representing 120 families and 286 genera. Proteobacteria held an overwhelming predominance with an average relative abundance of 58.67%, followed by Firmicutes (20.6%), Bacteroidetes (15.11%), Actinobacteria (3.13%), and Fusobacteria (1.1%). The remaining 14 phyla were only observed in a fraction of the samples with a combined average abundance of less than 1% (Fig. 1). Proteobacteria were abundant in ILD patients with a relative abundance of 65.71% compared to exacerbated COPD, stable COPD patients, and sarcoidosis patients, accounting for 56.54%, 59.68%, and 52.77%, respectively. In contrast, Firmicutes were abundant in stable COPD and ECOPD patients, (17.43% and 23.89% of the relative abundance), compared to ILD and sarcoidosis patients (Supplementary Table S4). At the family level, we observed the difference between the disease groups (Table 2 & Supplementary Table S5). The most abundant genera in the four disease groups were visualized in a heat map (Fig. 2). A range of genera showed relatively lower abundance but had high prevalence. These included *Serratia*, *Prevotella*, *Streptococcus*, *Reyranella*, *Escherichia*-*Shigella*, *Neisseria*, and *Ralstonia* (Fig. 2). *Escherichia*-*Shigella, Haemophilus*, *Pseudomonas,* and *Serratia* showed high variability among the four disease groups. *Serratia* was the most common genus in all groups except stable COPD. *Escherichia*-*Shigella* was also consistent among COPD groups along with *Pseudomonas*. *Enterobacter* was differentially abundant in stable COPD patients while *Klebsiella* and *Staphylococcus* were abundant in ILD patients.

**Table 2:** The five families most commonly identified from each disease groups and their percentage.

Family	Exacerbation COPD	Family	ILD	Family	Stable COPD	Family	Sarcoidosis
*Enterobacteriaceae*	19.53	*Enterobacteriaceae*	22.98	*Pasteurellaceae*	12.61	*Prevotellaceae*	13.43
*Streptococcaceae*	11.69	*Pasteurellaceae*	15.36	*Reyranellaceae*	11.90	*Burkholderiaceae*	12.09
*Prevotellaceae*	8.02	*Prevotellaceae*	8.30	*Prevotellaceae*	10.92	*Streptococcaceae*	11.34
*Pseudomonadaceae*	7.96	*Staphylococcaceae*	7.77	*Burkholderiaceae*	10.10	*Enterobacteriaceae*	10.14
*Pasteurellaceae*	7.81	*Streptococcaceae*	7.34	*Enterobacteriaceae*	9.61	*Reyranellaceae*	8.62

### Alpha diversity among disease groups

**Figure 3:**
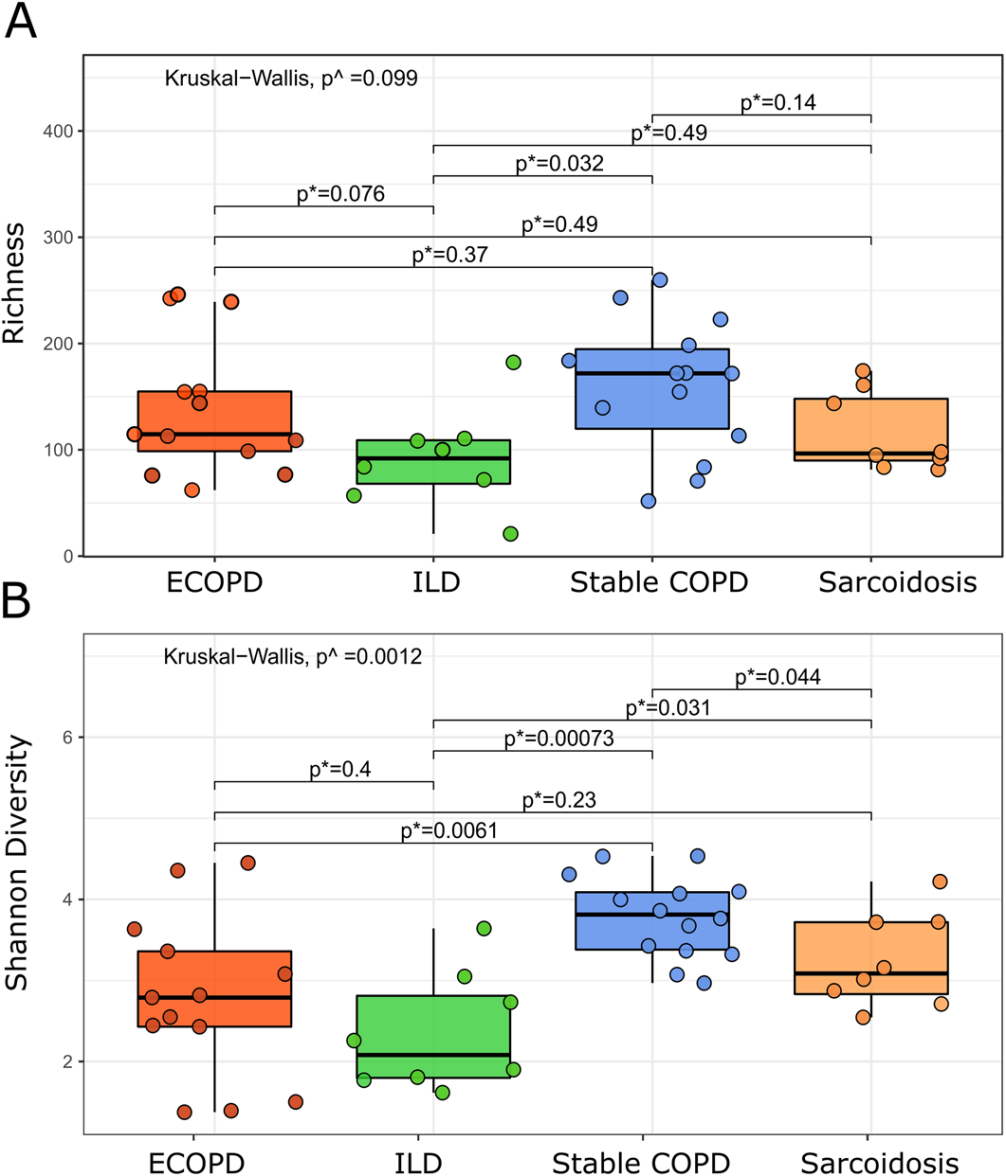
Observed richness (**A**) and Shannon diversity index (**B**). Comparing two groups using Mann–Whitney test; ^comparing two or more groups using Kruskal–Wallis test. *p* < 0.05 denotes statistical significance.

We found no significant difference in observed richness between the diseases (Fig. 3A; *p* = 0.099, Kruskal–Wallis test). However, using Shannon diversity index, we observed statistically significant differences between diseases (Fig. 3B; *p* = 0.001, Kruskal–Wallis test) with post hoc tests revealing higher diversity in stable COPD compared to ILD (*p* = 0.037), and sarcoidosis (*p* = 0.004) respectively; Mann–Whitney test, Bonferroni adjustment).

### Beta diversity among disease groups

**Figure 4:**
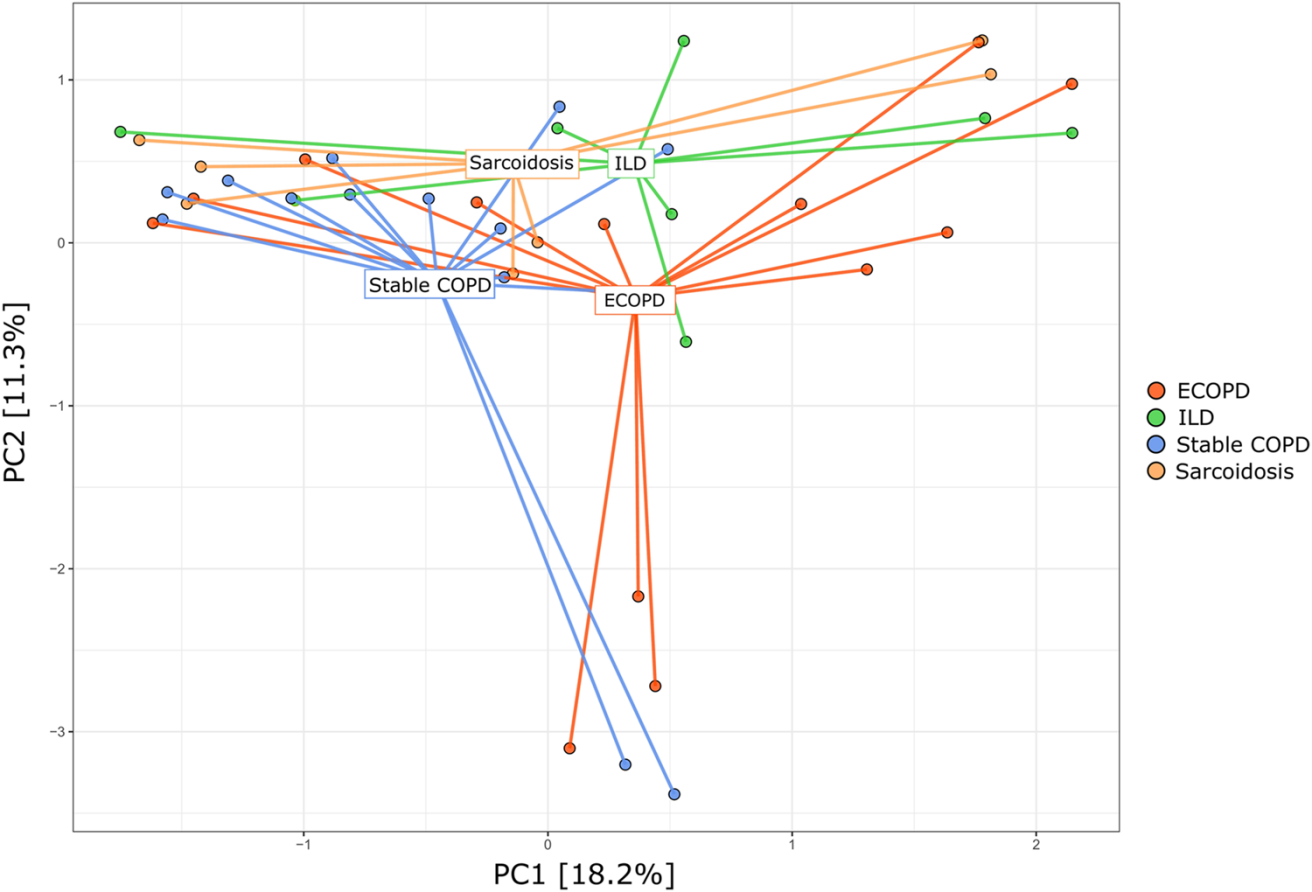
Principal component analysis. Dots represent samples and color represents different disease groups. First two principal components (PC) explained 29.5% of the variance.

Bray–Curtis based PCA plots were analyzed at the genus level to understand the community ordination (Fig. 4). This approach revealed extensive overlap in membership between the bacterial communities of the ECOPD, stable COPD, ILD, and sarcoidosis disease groups. The first two principal components accounted for 29.5% of variance explained, but we did not observe clear clustering. The PERMANOVA test was used to assess how much the overall variation could be explained in groups, indicating no notable separation among the groups (*p* = 0.0610).

### Microbial taxa associated with disease groups

**Figure 5:**
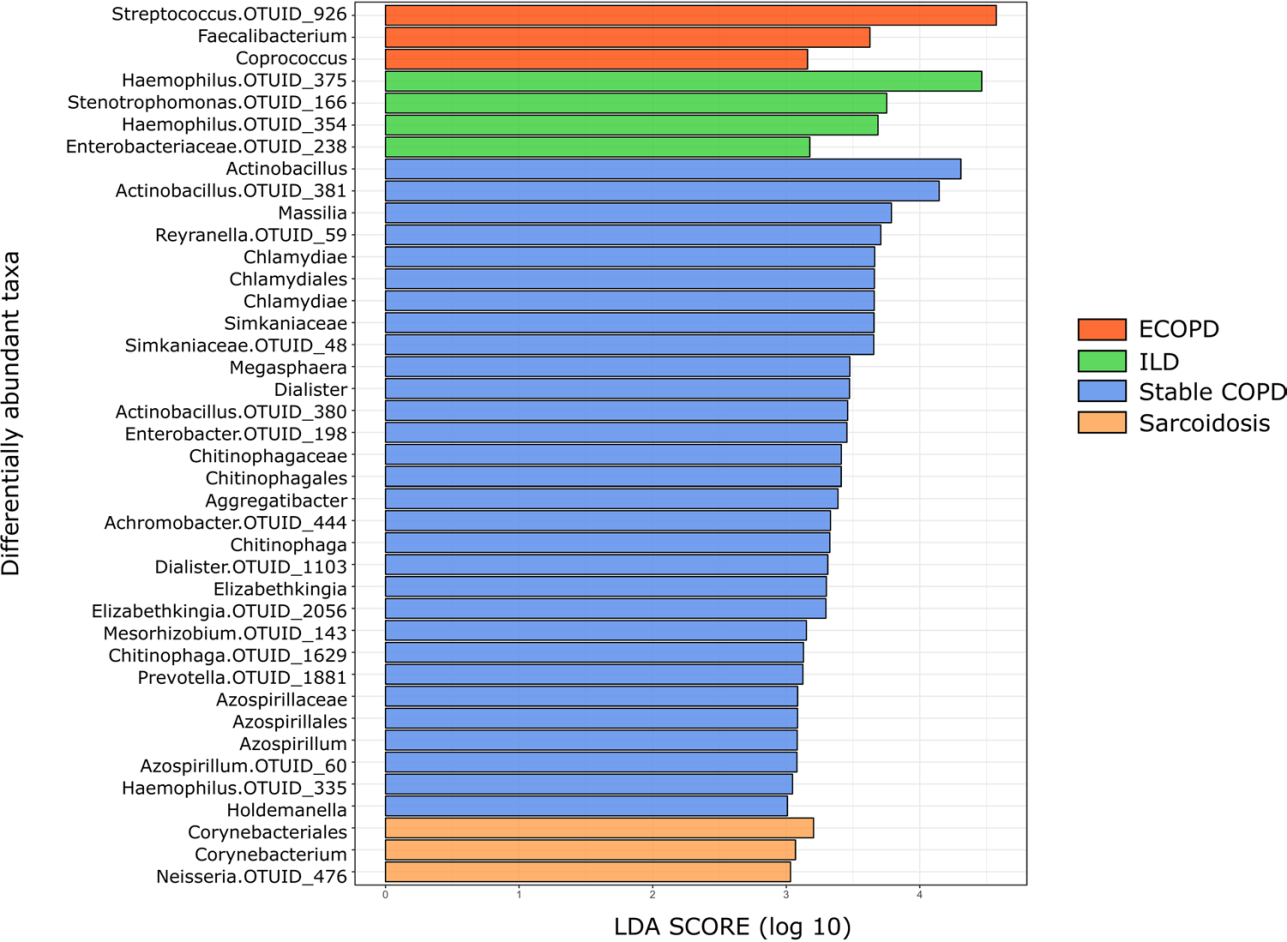
LDA shows distinct lung microbiome composition associated with ECOPD, stable COPD, ILD and sarcoidosis. LDA scores as calculated by LEfSe of taxa differentially abundant in different disease group. Only taxa with LDA scores of more than three and *p* value < 0.05 are shown here.

LEfSe identified 40 discriminative features, out of which, thirty taxa were discriminative for stable COPD patients, four taxa for ILD patients, and three taxa for ECOPD and sarcoidosis patients (Fig. 5). Taxa belonging to Firmicutes were significantly more abundant (*p* < 0.05) among ECOPD patients. Proteobacteria were more abundant among ILD patients, while Actinobacteria and Proteobacteria were significantly more abundant (*p* < 0.05) in sarcoidosis patients. On the other hand, Chlamydiae along with Firmicutes and Proteobacteria were significantly more abundant (*p* < 0.05) in ECOPD patient's lungs (Supplementary Table S6). In ECOPD patients, the microbiome was characterized by a preponderance of *Streptococcus* (LDA score [log10] > 4), whereas in the stable COPD patients, there was a preponderance of *Actinobacillus* (LDA score [log10] > 4). However, ILD patient`s microbiome showed a very high abundance of *Haemophilus* (LDA score [log10] > 4), while *Corynebacterium* was abundant in the sarcoidosis patient`s (LDA score [log10] > 3).

### Functional annotation of the lung microbiome

Predicated phenotypes based on taxonomic classification indicated that the majority of microbes were mesophilic (> 65%), gram-negative (> 74%) *Bacillus* (> 62%). A majority of the lung microbes were generally considered to be associated with humans (> 60%), while the remaining microbes were not commonly associated with a specific environment (> 30%). The majority of the identified lung microbes predict a higher potential for the onset of various human disorders. The percentage of such microbes was higher in the samples from sarcoidosis and other ILD groups (> 71–85%) as compared with the COPD group (62–70%). The majority of the microbes from COPD lung were found to play a significant role in ammonia oxidation, sulfur metabolism, and complex carbohydrate catabolism. Lung microbes in sarcoidosis and other ILD groups seemed to play a significant metabolic role in polyphenol metabolism and dehalogenation reactions in addition to the function carried out by COPD inherent microbes.

### Core microbiome and its association with diseases

Common members of a microbial community often perform moderate functioning of the host-microbial symbiotic system. We estimated a high degree of similarity between the core microbiome for each of the four diseases. Ten ASVs were shared among all of them; these belonged to the genera *Reyranella*, *Ochrobactrum*, *Mesorhizobium*, *Ralstonia*, *Achromobacter*, *Pseudomonas*, *Streptococcus*, *Granulicatella*, and two unclassified genera belonging to *Xanthobacteraceae*. Moreover, there were 11 ASVs unique to patients with sarcoidosis, three to ECOPD, 12 ASVs to stable COPD, and only two ASVs to ILD (Supplementary Table S7, Supplementary Fig. S3).

**Figure 6:**
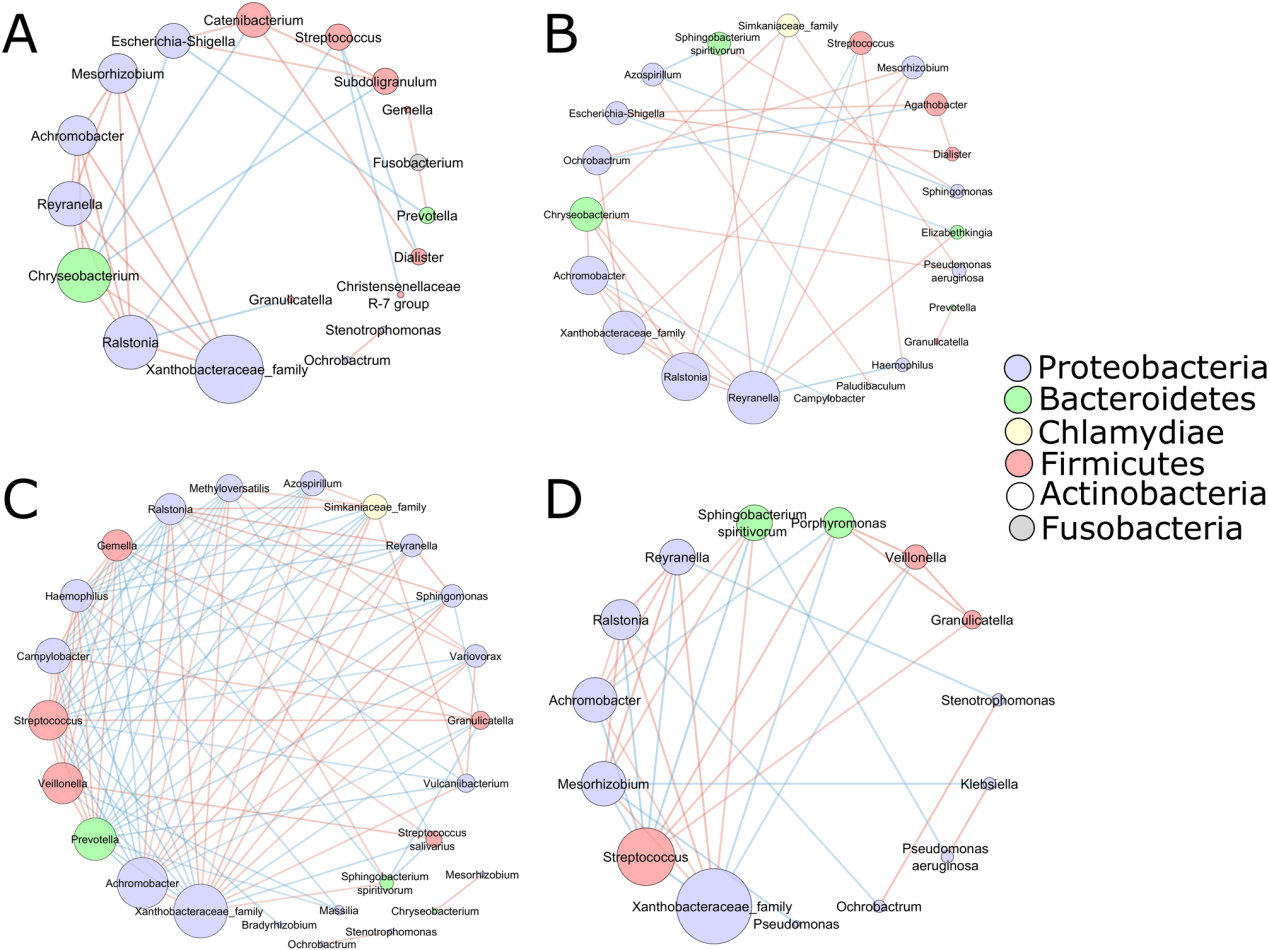
Bacterial co-existence and co-exclusion relationships with ASVs and different diseases. Each node represents ASVs. Each edge represents a significant correlation colored by co-existence (orange) or co-exclusion relationships (blue). The size of the node corresponds to its degree of connectivity, while edge lengths are arbitrary. ECOPD (**A**), stable COPD (**B**), sarcoidosis (**C**), and ILD (**D**).

To gain insight into the interaction between bacterial species in the lung microbiome, we performed a species network analysis (only correlations with an absolute value of 0.60, *p* < 0.05). Examination of the microbial network revealed that *Xanthobacteraceae* were highly connected with multiple other ASVs among all the disease groups (Fig. 6).

In ECOPD patients *Escherichia*-*Shigella* (Fig. 6A) positively correlated with *Subdoligranulum*, *Catenibacterium*, and negatively correlated with *Chryseobacterium* and *Prevotella*. Conversely, one of the most abundant genera i.e., *Streptococcus* showed a negative correlation with *Dialister*, *Ralstonia,* and *Christensenellaceae* R-7 groups. However, the rest of the most abundant genera i.e., *Serratia*, *Haemophilus,* and *Pseudomonas* did not correlate with any other genera. In the stable COPD subjects (Fig. 6B), the most abundant genus was *Reyranella*, which was negatively correlated with *Haemophilus* and *Streptococcus* whereas positively correlated with seven other genera that belong to *Achromobacter*, *Chryseobacterium*, *Mesorhizobium*, *Elizabethkingia*, *Sphingobacterium*, *Ralstonia*, *Xanthobacteraceae* family. The other two most abundant genera were *Escherichia*-*Shigella* and *Haemophilus*, both of which belong to the Proteobacteria phylum. *Escherichia*-*Shigella* showed a strong positive correlation with *Dialister* and *Agathobacter,* whereas it was negatively correlated with *Elizabethkingia*. On the other hand, *Haemophilus* showed no correlation with any other genera.

The sarcoidosis disease group (Fig. 6C) had the most negative connections with other members of the microbiota. Most abundant genera belonged to *Serratia*, *Prevotella*, *Streptococcus*, *Reyranella,* and *Ralstonia*. Except for the *Serratia*, which did not show any correlation, all other dominant genera showed a strong correlation with other genera in the group. *Prevotella* and *Streptococcus* genera were mostly negatively correlated, whereas *Reyranella* and *Ralstonia* showed a positive correlation.

In the ILD subjects (Fig. 6D), the most abundant genus, *Hemophilus*, did not show any correlation with others. Moreover, *Streptococcus* sp. showed correlation with 11 out of 14 genera in this group. It was found to be positively correlated with three genera and negatively correlated with eight different genera. Despite the abundance of the *Klebsiella* genera, it was positively correlated only with *Pseudomonas aeruginosa* and negatively correlated with *Mesorhizobium* sp.

## Discussion

The present results confirm earlier reports that the human respiratory tract contains a diverse microbiome^23^. Lung microbiome composition is influenced by the onset of respiratory illness, as well as with the usage of steroids, aerosols, and antibiotics^24,25^. During airway diseases, lung microbiome can exacerbate the diseases, leading to increased levels of morbidity and mortality^15^. Using the NGS platform, diverse non-cultivable bacteria were found in the respiratory tract^10^. This study has explored the alveolar microbiome of the patients with COPD (stable and exacerbation), ILD, and sarcoidosis to understand the similarities & differences between the disease-associated microbial phylotypes. We have accessed the composition, diversity, and core microbiome for each disease and identified which aspects are related to lung diseases in general and which are diseases specific. Additionally, to our knowledge, this is the first alveolar microbiome report among the Indian population.

It was found that the microbial members of the bronchial microbiome do not change significantly in COPD patients^26,27^. However, the predominance of Proteobacteria in the present study is in line with previous studies^14,15,17,18^. We observed a higher abundance of Firmicutes in stable COPD and ECOPD subjects compared to ILD and sarcoidosis groups. We found a higher alpha diversity in the stable COPD group in comparison to the other groups. A similar observation was found in previous studies^14,28^. Three taxa show a significant abundance in ECOPD patients- *Streptococcus*, *Faecalibacterium*, and *Coprococcus*. *Streptococcus* is the most widely recognized microbe found in COPD patients^14,17^. *Faecalibacterium* is a common resident of the human gut, while *Coprococcus* is usually found in the sputum, however, what role the latter two play in humans in the human lung is still unexplored.

Few efforts have been made to explore lung microbiome in the patients with sarcoidosis and other types of ILD^23,29^ and these studies were unable to differentiate the lung microbiome structure among these disease subtypes^23^. We were able to identify differences in their microbial composition. We found an increase in Actinobacteria and a decrease in Proteobacteria in sarcoidosis patients as compared to ILD subjects. The increased relative abundance of *Streptococcus* and *Staphylococcus* has been reported to contribute to disease progression in idiopathic pulmonary fibrosis^29^. Similarly, we also observed a higher relative abundance of *Streptococcus* and *Staphylococcus* in the ILD groups, as well as an increased alpha diversity in sarcoidosis compared to ILD patients. Besides, PCA showed differences in microbial variation between COPD (stable and exacerbation), ILD, and sarcoidosis patients. Moreover, current data show a significantly higher abundance of taxa belonging to the genera *Haemophilus, Stenotrophomonas,* and *Enterobacteriaceae* family in the ILD group, whereas *Corynebacterium* and *Neisseria* are more abundant in the sarcoidosis group. However, *Haemophilus*, known as pathogenic-bacteria, is usually observed in COPD patients^30^.

ILD groups have enriched unique microbial groups. Moreover, we also observed a significantly higher abundance of *Haemophilus* in stable COPD groups. These deviations could be seen as a possible outcome of diverse ethnicity, as commonly observed in other human microbiome studies^31,32^.

When we compared the core microbiome of COPD (stable and exacerbation), ILD, and sarcoidosis patients, we observed that eleven taxa were shared among these disease groups. This supports the idea that many features shared between microbiota differ compositionally between these disease groups. Additionally, the core alveolar microbiome in respiratory illness was found altered as compared to that of a healthy lung microbiome. Core lung microbiome of a healthy individual harbors nine microbial genera^33,34^ of which only *Pseudomonas*, *Streptococcus*, *Prevotella*, and *Sphingobacterium* were shared within the studied core microbiomes. This result supports the hypothesis that the microbiome biotransformation may lead to the onset of pathophysiological disorders^35^.

Furthermore, in the genus-level abundance network analysis, *Xanthobacteraceae* were highly connected with multiple other nodes between all the disease groups, indicating it as a keystone microbial taxon. Most of the genera in stable COPD, ECOPD, ILD, and sarcoidosis patients showed both positive and negative correlation; however, ECOPD and stable COPD patients showed a more positive correlation. We also noticed many potential clinically relevant taxa such as *Streptococcus* sp. (observed in all diseased groups), *Haemophilus sp.* (observed in stable COPD patients), *Escherichia*-*Shigella sp.* (observed in stable and ECOPD patients), and *Pseudomonas aeruginosa* (observed in stable COPD and ILD patients), show correlation with other taxa^14–22^. However, correlations between taxa are not proof of functional relationships between members of the community. Therefore, further studies are required to focus on the functional role of such taxa found within these communities.

The present study has used very stringent inclusion criteria for patients screening. Though it has allowed the identification of unbiased disease-specific samples, but also reduced the number of samples (~ 50 fold) for the downstream analysis. Due to the limitation of samples, the current study is slightly underpowered and higher numbers of samples are required to statistically strengthen the proposed claims. However, this pilot study provides preliminary evidence in support of the hypothesis that there are diseases specific differences in the microbiomes of COPD (stable and exacerbation), ILD, and sarcoidosis patients.

Moreover, our study adds further insights into the microbial composition of the lung microbiota of Indian patients suffering from COPD, ILD, or sarcoidosis. Each disease subtype has differential microbial phylotypes that correlate with the abundance profile of other microbial taxa to possibly remodel the lung microbiome structure. This study enhances our understanding of lung microbial ecology in various respiratory illnesses. Identified microbial signatures could be utilized as prognostic markers for respiratory disease diagnosis and therapeutics.

## Methods

### Patient recruitment and Broncho-Alveolar Lavage (BAL) sample collection

This study was approved by the institutional human ethics committee, Vallabhbhai Patel Chest Institute, University of Delhi, Delhi, India. Adult patients with stable COPD, both male and female with a history of smoking (> 10 pack-years) and/or biomass fuel exposure (> 10 years) attending Vallabhbhai Patel Chest Institute were invited to participate in the study. Patients classified as having exacerbated COPD if they presented within increased cough or sputum production. All patients suspected of ILD underwent clinical evaluation including detailed history and examination. The diagnosis of ILD was made based on the American Thoracic Society/European Respiratory Society International Multidisciplinary Consensus Classification of Idiopathic Interstitial Pneumonia 2001 guidelines^36^. Similarly, the diagnosis of sarcoidosis was made based on the compatible clinical, radiological, laboratory, and where available, histopathological parameters, as per the joint statement of the American Thoracic Society, the European Respiratory Society, and the World Association of Sarcoidosis, and Other Granulomatous Disorders (ATS/ERS/WASOG), and also the simultaneous exclusion of any other cause of the granulomatous disorder^37^. Since Bronchoscopy was used as a diagnostic criterion, patients were excluded if they have taken antibiotics or steroids prior to their inclusion.

After providing written informed consent, the patients underwent bronchoscopy as per the British Thoracic Society (BTS) guidelines for bronchoscopy 2013. This study included bronchoalveolar lavage collected from ECOPD (n = 13), stable COPD (n = 14), ILD (n = 8), and sarcoidosis (n = 8) patients, as well as saline buffer passed through a bronchoscope to be used as negative control.

### Metagenomic DNA isolation from BAL samples and 16S rRNA gene sequencing

Each BAL sample (1.5 ml) was centrifuged at 13,000 rpm for 1 min to collect the bacterial pellet. The bacterial pellet was processed with the alkaline lysis method^38^. Metagenomic DNA quantification was performed with Qubit 2.0 using high sensitivity DNA quantification kit (Invitrogen, USA). All samples were diluted to a DNA concentration of 25 ng μl^-1^. The V3-V4 region of the bacterial 16S rRNA gene was amplified using gene-specific primer sequences (Fwd 5′-TCGTCGGCAGCGTCAGATGTGTATAAGAGACAGCCTACGGGNGGCWGCAG-3` and Rev 5′-GTCTCGTGGGCTCGGAGATGTGTATAAGAGACAGGACTACHVGGGTATCTA ATCC-3`)^39^. Nextera XT Index kit (Illumina, USA) was used to index each sample during library preparation following Illumina technology workflow document (www.support.illumina.com). The indexed 16S rRNA amplicons were pooled in equimolar concentration followed by paired-end sequencing on the Illumina MiSeq platform using paired-end MiSeq 600 cycle V3 sequencing Kit following manufacturer instructions^39^.

### Sequence and statistical analyses

Primers were removed from the MiSeq demultiplex FASTQ using “cutadapt"^40^. Further, reads were analyzed by the QIIME2 pipeline^41^ through dada2^42^ to infer the presence and relative abundance of amplicon sequence variants (ASVs) across the samples. Based on data-derived rates of Illumina sequencing errors, dada2 estimated an abundance distribution of distinct ASVs, which may differ by only a single nucleotide. Using read quality scores for the dataset, forward and reverse reads were truncated at 270 bp and 200 bp, followed by trimming the 5`-end till 6 bp for both forward and reverse reads, respectively; other quality parameters used dada2 default values. Taxonomy was assigned using a pre-trained Naïve Bayes classifier (Silva database, release 132)^43^. The rarefaction curves (Supplementary Fig. S1) show the observed richness and Shannon diversity. To avoid the bias due to sampling depth, we rarefied our dataset to 4351 high-quality sequences per sample (90% of the minimum sample reads) using an in-house script. The function rarefies each sample 100 times, calculates the mean and standard deviation for observed richness and Shannon diversity index, and returns the ASV counts for the iteration with the lowest mean Bray–Curtis distance among the 100 iterations.

All downstream analyses were performed on this rarefied ASVs table unless otherwise mentioned. We used two diversity indices i.e., observed richness, the number of taxa present in a sample at a particular taxonomic level, and Shannon diversity index, a composite measure of both species richness and evenness. Alpha and beta diversity was calculated using phyloseq v1.20.0^44^ and visualized with ggplot2 v2.2.1in R v3.4.1.^45^. Comparison of community richness and diversity between the four disease groups was assessed by the Kruskal–Wallis test, with post hoc tests, performed using the Mann–Whitney test with Bonferroni adjustment applied. Significance testing between the disease groups for beta diversity was assessed using the PERMANOVA (permutational multivariate analysis of variance). LEfSe was used to identify the microbiological markers associated with stable COPD, ECOPD, ILD, and sarcoidosis disease groups by linear discriminate analysis (LDA) effect size of 3, and for multiclass analysis one-against-all option was used with default parameters^46^.

### Functional annotation of the lung microbiome

The taxonomically affiliated OTU table was used to annotate physiological functions and the lifestyle of human lung microbes associated with various disease subsets with the METAGENassist server^47^.

### Core microbiota and bacterial co-occurrence

Considering the variable nature of metagenomic compositional data, we performed further analysis only for conserved taxa. Towards this, we estimated core microbiota within the samples with a presence in at least 50% of the samples within each disease group on the non-rarefied data. We examined co-occurrence patterns using network analysis on the core microbiota using Sparse Correlations for Compositional data algorithm (SparCC) with a bootstrap procedure repeated 100 times^48^. Co-occurrence was considered robust when the correlations (either positive or negative) were both ≥ 0.6 and correlation coefficients with two-tailed *p* values smaller than 0.05. The correlation was imported into Cytoscape v3.6.0 to build the co-occurrence network, where each node represents taxa and the edges between the nodes represent the correlation coefficients between taxa^49^.

### Ethics approval and consent to participate

This study was carried out by following the recommendations of the Indian Council of Medical Research, India guidelines for biomedical research, with written informed consent from all subjects. All subjects gave written informed consent under the Declaration of Helsinki. The protocol was approved by the Institutional human ethics committee, Vallabhbhai Patel Chest Institute, University of Delhi, Delhi, India.

### Consent for publication

The manuscript has been read and approved for submission by the named authors.

## Supplementary information

Supplementary information.

## Data Availability

Sequence data generated in this study have been deposited at NCBI with an SRA submission ID SUB4935309 and Bio project accession ID PRJNA512576.
