# Supplementary material for: Comparative analysis of the alveolar microbiome in COPD, ECOPD, Sarcoidosis, and ILD patients to identify respiratory illnesses specific microbial signatures
Source: Sci Rep. 2021 Feb 17;11:3963. doi: 10.1038/s41598-021-83524-2 (PMC7889618; doi:10.1038/s41598-021-83524-2)
Supplement: Supplementary file 1 — Supplementary information. [file 41598_2021_83524_MOESM1_ESM.pdf]

Comparative analysis of the alveolar microbiome in COPD, ECOPD, Sarcoidosis, and ILD patients to identify respiratory illnesses specific microbial signatures

Shashank Gupta[1,6]¶, Malini Shariff[2]¶, Gaura Chaturvedi[3,4]¶, Agrima Sharma[2], Nitin Goel[5], Monika Yadav[6], Martin S. Mortensen[1], Søren J. Sørensen[1], Mitali Mukerji[3], Nar Singh Chauhan[6*]

¶ Authors have contributed equally

[1]Section of Microbiology, Department of Biology, University of Copenhagen, 2100 Copenhagen, Denmark

[2]Department of Microbiology, Vallabhbhai Patel Chest Institute, University of Delhi, Delhi-110007, India.

[3] Genomics and Molecular Medicine, CSIR-Institute of Genomics and Integrative Biology (CSIR-IGIB), South Campus, Sukhdev Vihar, Mathura Road, New Delhi- 110025, India

[4]Academy of Scientific and Innovative Research (AcSIR), Ghaziabad- 201002, India

[5]Department of Pulmonary Medicine, Vallabhbhai Patel Chest Institute, University of Delhi, Delhi-110007, India.

[6]Department of Biochemistry, Maharshi Dayanand University, Rohtak -124001, Haryana, India.

*Corresponding Author

Email: nschauhan@mdurohtak.ac.in

(A) Supplementary Tables

Supplementary Table S1: Metadata of the patients with sarcoidosis enrolled in present study.

Sample ID	Gender (M/F)*	AGE (Years)	Physiological Symptoms	Clinical Investigations	Diabetes	Heart disease	Others
C12	M	36	Cough with sputum, Breathlessness, Repeated cold, Fever	Granulomatous inflammation, Bilateral Hilar Lymphadenopathy- Stage 2	No	No	No Hypertension, No Irritable bowel syndrome (IBS)
C16	M	65	Cough Breathlessness, Repeated Cold	Granulomatous inflammation , Bilaterally symmetrical Hilar Lymphadenopathy	No	No	No information available
C23	F	45	Cough, Breathlessness, Pain in abdomen, Fever, Repeated Cold	Granulomatous inflammation , Bilateral Hilar Lymphadenopathy	No	No	No Hypertension, No Irritable bowel syndrome (IBS)
C27	F	56	Breathlessness, Cough	Granulomatous inflammation, Bilaterally symmetrical Hilar Lymphadenopathy	No	No	No Hypertension, No Irritable bowel syndrome (IBS)
C32	M	30	Cough Breathlessness, Fever	Granulomatous inflammation, Bilaterally symmetrical Hilar Lymphadenopathy	No	No	No Hypertension, No Irritable bowel syndrome (IBS)
C57	M	42	Breatlessness, Cheat Pain,	Granulomatous inflammation, Bilateral Hilar Lymphadenopathy- Stage 1	No	No	No Hypertension, No Irritable bowel syndrome (IBS)
C62	F	54	Breatlessness, Chest Pain	Granulomatous inflammation, Right Para tracheal and bilateral Hilar Lymphadenopathy -Stage1	No	No	No Hypertension, No Irritable bowel syndrome (IBS)
C67	M	28	Breathlessness, Cough	Granulomatous inflammation , Mediastinal lymphadenopathy	No	No	No information available

* Here M: Male, F: Female.

Supplementary Table S2: Metadata of patients with interstitial lung disorder enrolled in present study.

Sample ID	Age (Years)	Gender* (M/F)	Physiological symptoms	Pathological Investigations	Proposed Diagnosis	Diabetes	Heart disease	Others
C3	35	F	Cough, breathlessness	Both lung fields show fibrosis and traction, Chronic interstitial thickening, Honey comb ground glass opacities	Idiopathic pulmonary fibrosis	No	No	No Hypertension, No irritable bowel syndrome (IBS)
C13	55	F	Cough with sputum, Breathlessness, Repeated cold	Bilateral lower lobe reticular opacities more on right side, Ectatic changes in all lobes of both lungs	Respiratory bronchiolitis	No	No	No Hypertension, No irritable bowel syndrome (IBS)
C14	52	M	Breathlessness, Cough	Bilateral lower lobe ground glass opacities and reticular shadows	Nonspecific interstitial pneumonia	No	No	No Hypertension, No irritable bowel syndrome (IBS)
C19	55	F	Breathlessness, Cough, Fever, Wt Loss	Patchy consolidation and multiple nodules in right upper and middle lobes and bilateral lower lobes, Subcentimetric Mediastinal lymphadenopathy	Nonspecific interstitial pneumonia	No	No	No Hypertension, No irritable bowel syndrome (IBS)
C21	65	M	Cough, Breathlessness	Bilateral cavities in upper lobes and centrilobular nodules	Respiratory bronchiolitis	No	No	No Hypertension, No irritable bowel syndrome (IBS)
C50	44	F	Cough, breathlessness	Bilateral mid zone and lower zone non homogeneous opacities with areas of air bronchogram with right costophrenic angle blunting	Unclassifiable ILD	No	No	No Hypertension, No irritable bowel syndrome (IBS)
C58	70	M	Breathlessness, Cough	Both lung fields show fibrosis and traction, Chronic interstitial thickening, honey comb ground glass opacities	Idiopathic pulmonary fibrosis	No	No	No Hypertension, No irritable bowel syndrome (IBS)
C64	52	F	Breathlessness, cough	Bilateral lower lobe ground glass opacities and reticular shadows	Nonspecific interstitial pneumonia	No	No	No Hypertension, No irritable bowel syndrome (IBS)

* Here M: Male, F: Female.

Supplementary Table S3: Metadata of patients with COPD enrolled in present study.

Sample ID	Gender	Smoker (Active:1; Inactive: 0)	Biomass Exposure (Yes: 1; No:0)	Diseases Diagnosis	Other Information
T14	M	0	NA	Stable COPD	No Hypertension, No irritable bowel syndrome (IBS)
T18	M	1	0	Stable COPD	No Hypertension, No irritable bowel syndrome (IBS)
T19	M	1	0	Stable COPD	No Hypertension, No irritable bowel syndrome (IBS)
T20	M	1	0	Stable COPD	No Hypertension, No irritable bowel syndrome (IBS)
T21	M	0	NA	Stable COPD	No information available
T22	M	1	0	Stable COPD	No Hypertension, No irritable bowel syndrome (IBS)
T24	M	1	0	Stable COPD	No Hypertension, No irritable bowel syndrome (IBS)
T27	M	1	0	Stable COPD	No Hypertension, No irritable bowel syndrome (IBS)
T43	M	1	0	Stable COPD	No Hypertension, No irritable bowel syndrome (IBS)
T45	M	1	0	Stable COPD	No Hypertension, No irritable bowel syndrome (IBS)
T46	M	1	0	Stable COPD	No Hypertension, No irritable bowel syndrome (IBS)
T47	M	1	0	Stable COPD	No Hypertension, No irritable bowel syndrome (IBS)
T48	M	NA	NA	Stable COPD	No information available
T49	M	0	0	Stable COPD	No Hypertension, No irritable bowel syndrome (IBS)
T1	F	0	1	Exacerbated COPD (ECOPD)	No Hypertension, No irritable bowel syndrome (IBS)
T11	F	0	1	Exacerbated COPD (ECOPD)	No Hypertension, No irritable bowel syndrome (IBS)
T12	M	1	0	Exacerbated COPD (ECOPD)	No information available
T29	M	1	0	Exacerbated COPD (ECOPD)	No information available
T36	M	0	1	Exacerbated COPD (ECOPD)	No information available
T37	M	1	0	Exacerbated COPD (ECOPD)	No Hypertension, No irritable bowel syndrome (IBS)
T38	M	1	NA	Exacerbated COPD (ECOPD)	No information available
T4	M	1	0	Exacerbated COPD (ECOPD)	No Hypertension, No irritable bowel syndrome (IBS)
T42	M	1	1	Exacerbated COPD (ECOPD)	No Hypertension, No irritable bowel syndrome (IBS)
T5	M	0	NA	Exacerbated COPD (ECOPD)	No information available
T6	M	1	0	Exacerbated COPD (ECOPD)	No Hypertension, No irritable bowel syndrome (IBS)
T7	M	1	0	Exacerbated COPD (ECOPD)	No information available
T8	F	0	1	Exacerbated COPD (ECOPD)	No Hypertension, No irritable bowel syndrome (IBS)

* Here M: Male, F: Female.

Supplementary Table S4: List of bacteria at phylum level and their abundance observed by sequencing.

Phylum	Diseases	Abundance
Acidobacteria	Exacerbated COPD	0.007071761
Actinobacteria	Exacerbated COPD	4.946697
Armatimonadetes	Exacerbated COPD	0.005303821
Bacteroidetes	Exacerbated COPD	12.70265
Chlamydiae	Exacerbated COPD	0.2581193
Chloroflexi	Exacerbated COPD	0.01591146
Cyanobacteria	Exacerbated COPD	0.178562
Deinococcus-Thermus	Exacerbated COPD	0.005303821
Epsilonbacteraeota	Exacerbated COPD	0.1237558
Firmicutes	Exacerbated COPD	23.89371
Fusobacteria	Exacerbated COPD	0.8715945
Patescibacteria	Exacerbated COPD	0.07071761
Planctomycetes	Exacerbated COPD	0.00353588
Proteobacteria	Exacerbated COPD	56.53696
Spirochaetes	Exacerbated COPD	0.1538108
Synergistetes	Exacerbated COPD	0.007071761
Tenericutes	Exacerbated COPD	0.07248555
Verrucomicrobia	Exacerbated COPD	0.1202199
WPS-2	Exacerbated COPD	0
Acidobacteria	ILD	0.008618708
Actinobacteria	ILD	0.6751322
Armatimonadetes	ILD	0.008618708
Bacteroidetes	ILD	11.3336
Chlamydiae	ILD	0.2643071
Chloroflexi	ILD	0
Cyanobacteria	ILD	0.02585613
Deinococcus-	ILD	0.02872903

Thermus		
Epsilonbacteraeota	ILD	0.2815445
Firmicutes	ILD	20.69639
Fusobacteria	ILD	0.6866238
Patescibacteria	ILD	0.2413238
Planctomycetes	ILD	0.008618708
Proteobacteria	ILD	65.71191
Spirochaetes	ILD	0.002872903
Synergistetes	ILD	0
Tenericutes	ILD	0
Verrucomicrobia	ILD	0
WPS-2	ILD	0
Acidobacteria	Stable COPD	0.1625242
Actinobacteria	Stable COPD	1.397052
Armatimonadetes	Stable COPD	0.0476081
Bacteroidetes	Stable COPD	17.96139
Chlamydiae	Stable COPD	1.155728
Chloroflexi	Stable COPD	0.008208294
Cyanobacteria	Stable COPD	0.02626654
Deinococcus-Thermus	Stable COPD	0
Epsilonbacteraeota	Stable COPD	0.216699
Firmicutes	Stable COPD	17.42621
Fusobacteria	Stable COPD	1.094986
Patescibacteria	Stable COPD	0.4744394
Planctomycetes	Stable COPD	0.01477493
Proteobacteria	Stable COPD	59.67922
Spirochaetes	Stable COPD	0.06566635
Synergistetes	Stable COPD	0.01149161
Tenericutes	Stable COPD	0.01313327
Verrucomicrobia	Stable COPD	0.1674492

WPS-2	Stable COPD	0.003283317
Acidobacteria	Sarcoidosis	0.06320386
Actinobacteria	Sarcoidosis	5.656746
Armatimonadetes	Sarcoidosis	0.01149161
Bacteroidetes	Sarcoidosis	17.78901
Chlamydiae	Sarcoidosis	0.5831993
Chloroflexi	Sarcoidosis	0
Cyanobacteria	Sarcoidosis	0.005745806
Deinococcus-Thermus	Sarcoidosis	0.01723742
Epsilonbacteraeota	Sarcoidosis	0.1522638
Firmicutes	Sarcoidosis	20.69352
Fusobacteria	Sarcoidosis	1.887497
Patescibacteria	Sarcoidosis	0.2011032
Planctomycetes	Sarcoidosis	0.01149161
Proteobacteria	Sarcoidosis	52.7781
Spirochaetes	Sarcoidosis	0.07756838
Synergistetes	Sarcoidosis	0.005745806
Tenericutes	Sarcoidosis	0.04596644
Verrucomicrobia	Sarcoidosis	0
WPS-2	Sarcoidosis	0

Supplementary Table S5: List of bacteria at family level and their abundance observed by sequencing.

Family	Diseases	Abundance
Enterobacteriaceae	Exacerbated COPD	19.52689921
Streptococcaceae	Exacerbated COPD	11.68785248
Prevotellaceae	Exacerbated COPD	8.022912505
Pseudomonadaceae	Exacerbated COPD	7.957498718
Pasteurellaceae	Exacerbated COPD	7.814295564
Burkholderiaceae	Exacerbated COPD	4.838852253
Reyranellaceae	Exacerbated COPD	4.798189629
Neisseriaceae	Exacerbated COPD	4.532998603
Ruminococcaceae	Exacerbated COPD	4.357972526
Micrococcaceae	Exacerbated COPD	3.98670509
Xanthobacteraceae	Exacerbated COPD	2.975443311
Lachnospiraceae	Exacerbated COPD	2.584728533
Rhizobiaceae	Exacerbated COPD	2.201085515
Veillonellaceae	Exacerbated COPD	1.382529215
Weeksellaceae	Exacerbated COPD	1.235790181
Porphyromonadaceae	Exacerbated COPD	1.053692343
Lactobacillaceae	Exacerbated COPD	0.935240351
Xanthomonadaceae	Exacerbated COPD	0.869826565
Staphylococcaceae	Exacerbated COPD	0.827396001
Carnobacteriaceae	Exacerbated COPD	0.783197497
Fusobacteriaceae	Exacerbated COPD	0.698336368
Sphingobacteriaceae	Exacerbated COPD	0.655905804
Bacteroidaceae	Exacerbated COPD	0.565740855
Actinomycetaceae	Exacerbated COPD	0.486183548
Flavobacteriaceae	Exacerbated COPD	0.419001821
Sphingomonadaceae	Exacerbated COPD	0.415465941
Bacillales_Family_XI	Exacerbated COPD	0.339444513
Erysipelotrichaceae	Exacerbated COPD	0.327068932

Simkaniaceae	Exacerbated COPD	0.258119265
Christensenellaceae	Exacerbated COPD	0.249279564
Rikenellaceae	Exacerbated COPD	0.228064282
Tannerellaceae	Exacerbated COPD	0.190937539
Leptotrichiaceae	Exacerbated COPD	0.173258137
Coriobacteriaceae	Exacerbated COPD	0.160882556
Barnesiellaceae	Exacerbated COPD	0.159114616
Spirochaetaceae	Exacerbated COPD	0.153810795
Campylobacteraceae	Exacerbated COPD	0.123755812
Bifidobacteriaceae	Exacerbated COPD	0.120219932
Peptostreptococcaceae	Exacerbated COPD	0.113148171
Akkermansiaceae	Exacerbated COPD	0.10607641
Clostridiales_vadinBB60_group	Exacerbated COPD	0.10077259
Moraxellaceae	Exacerbated COPD	0.09723671
Hyphomicrobiaceae	Exacerbated COPD	0.072485547
Succinivibrionaceae	Exacerbated COPD	0.072485547
Beijerinckiaceae	Exacerbated COPD	0.068949667
Clostridiales_Family_XIII	Exacerbated COPD	0.068949667
Corynebacteriaceae	Exacerbated COPD	0.061877906
Rhodocyclaceae	Exacerbated COPD	0.058342026
Azospirillaceae	Exacerbated COPD	0.056574086
Chitinophagaceae	Exacerbated COPD	0.047734385
Mycoplasmataceae	Exacerbated COPD	0.047734385
Acidaminococcaceae	Exacerbated COPD	0.045966444
Lentimicrobiaceae	Exacerbated COPD	0.044198504
Atopobiaceae	Exacerbated COPD	0.038894684
Caulobacteraceae	Exacerbated COPD	0.037126744
Eggerthellaceae	Exacerbated COPD	0.037126744
Muribaculaceae	Exacerbated COPD	0.033590863
Aerococcaceae	Exacerbated COPD	0.030054983
Rhizobiales_Incertae_Sedis	Exacerbated COPD	0.030054983

Mycobacteriaceae	Exacerbated COPD	0.022983222
Bacillaceae	Exacerbated COPD	0.021215282
Marinifilaceae	Exacerbated COPD	0.017679402
Thermomicrobiales_JG30_KF_CM45	Exacerbated COPD	0.015911462
Devosiaceae	Exacerbated COPD	0.014143521
Chthoniobacteraceae	Exacerbated COPD	0.012375581
Rhodanobacteraceae	Exacerbated COPD	0.012375581
Streptomycetaceae	Exacerbated COPD	0.012375581
Rhodobacteraceae	Exacerbated COPD	0.010607641
Propionibacteriaceae	Exacerbated COPD	0.008839701
Saccharimonadaceae	Exacerbated COPD	0.008839701
Cardiobacteriaceae	Exacerbated COPD	0.007071761
Halomonadaceae	Exacerbated COPD	0.007071761
*Solibacteraceae_Subgroup_3_*	Exacerbated COPD	0.007071761
Synergistaceae	Exacerbated COPD	0.007071761
Bacteriovoracaceae	Exacerbated COPD	0.005303821
Clostridiales_Family_XI	Exacerbated COPD	0.005303821
Fimbriimonadaceae	Exacerbated COPD	0.005303821
Hydrogenophilaceae	Exacerbated COPD	0.005303821
Microbacteriaceae	Exacerbated COPD	0.005303821
Paludibacteraceae	Exacerbated COPD	0.005303821
Thermaceae	Exacerbated COPD	0.005303821
Alteromonadaceae	Exacerbated COPD	0.00353588
Hymenobacteraceae	Exacerbated COPD	0.00353588
Peptococcaceae	Exacerbated COPD	0.00353588
Pirellulaceae	Exacerbated COPD	0.00353588
Dermabacteraceae	Exacerbated COPD	0.00176794
Desulfovibrionaceae	Exacerbated COPD	0.00176794
Geodermatophilaceae	Exacerbated COPD	0.00176794
Leuconostocaceae	Exacerbated COPD	0.00176794
Nocardiaceae	Exacerbated COPD	0.00176794

Opitutaceae	Exacerbated COPD	0.00176794
Acetobacteraceae	Exacerbated COPD	0
Aeromonadaceae	Exacerbated COPD	0
Anaerolineaceae	Exacerbated COPD	0
Anaeroplasmataceae	Exacerbated COPD	0
Bacillales_Family_XII	Exacerbated COPD	0
Bacteroidales_F082	Exacerbated COPD	0
Betaproteobacteriales_TRA3_20	Exacerbated COPD	0
Blastocatellaceae	Exacerbated COPD	0
Candidatus_Saccharibacteria_bacterium_UB2523	Exacerbated COPD	0
Cellvibrionaceae	Exacerbated COPD	0
Chlamydiales_cvE6	Exacerbated COPD	0
Chromobacteriaceae	Exacerbated COPD	0
Coriobacteriales_Incertae_Sedis	Exacerbated COPD	0
Coxiellaceae	Exacerbated COPD	0
Enterococcaceae	Exacerbated COPD	0
Geminicoccaceae	Exacerbated COPD	0
Gemmataceae	Exacerbated COPD	0
Ilumatobacteraceae	Exacerbated COPD	0
Intrasporangiaceae	Exacerbated COPD	0
Kaistiaceae	Exacerbated COPD	0
Legionellaceae	Exacerbated COPD	0
Listeriaceae	Exacerbated COPD	0
Nocardioidaceae	Exacerbated COPD	0
Oligoflexales_0319_6G20	Exacerbated COPD	0
Paenibacillaceae	Exacerbated COPD	0
Parvibaculaceae	Exacerbated COPD	0
Planococcaceae	Exacerbated COPD	0
Pseudonocardiaceae	Exacerbated COPD	0
Rickettsiaceae	Exacerbated COPD	0
Rubinisphaeraceae	Exacerbated COPD	0

Sneathiellaceae	Exacerbated COPD	0
Sphingobacteriales_NS11_12_marine_group	Exacerbated COPD	0
Thermoactinomycetaceae	Exacerbated COPD	0
Enterobacteriaceae	ILD	22.98034934
Pasteurellaceae	ILD	15.35853827
Prevotellaceae	ILD	8.296943231
Staphylococcaceae	ILD	7.774074925
Streptococcaceae	ILD	7.343139508
Pseudomonadaceae	ILD	6.613422202
Reyranellaceae	ILD	6.357733854
Burkholderiaceae	ILD	5.639508159
Xanthobacteraceae	ILD	3.335440129
Veillonellaceae	ILD	3.145828545
Rhizobiaceae	ILD	1.990921627
Xanthomonadaceae	ILD	1.192254654
Neisseriaceae	ILD	1.091703057
Porphyromonadaceae	ILD	0.973914043
Weeksellaceae	ILD	0.922201793
Ruminococcaceae	ILD	0.781429556
Sphingobacteriaceae	ILD	0.76419214
Lachnospiraceae	ILD	0.634911515
Fusobacteriaceae	ILD	0.476901862
Sphingomonadaceae	ILD	0.43380832
Campylobacteraceae	ILD	0.281544473
Carnobacteriaceae	ILD	0.281544473
Micrococcaceae	ILD	0.267179959
Simkaniaceae	ILD	0.264307056
Saccharimonadaceae	ILD	0.22695932
Leptotrichiaceae	ILD	0.209721903
Moraxellaceae	ILD	0.195357389
Erysipelotrichaceae	ILD	0.192484486

Bacillales_Family_XI	ILD	0.149390945
Flavobacteriaceae	ILD	0.12353482
Bacillaceae	ILD	0.112043208
Tannerellaceae	ILD	0.112043208
Actinomycetaceae	ILD	0.106297403
Hyphomicrobiaceae	ILD	0.106297403
Azospirillaceae	ILD	0.1034245
Bacteroidaceae	ILD	0.091932889
Caulobacteraceae	ILD	0.091932889
Coriobacteriaceae	ILD	0.086187083
Beijerinckiaceae	ILD	0.074695472
Corynebacteriaceae	ILD	0.074695472
Bifidobacteriaceae	ILD	0.057458056
Atopobiaceae	ILD	0.054585153
Christensenellaceae	ILD	0.045966444
Acidaminococcaceae	ILD	0.040220639
Clostridiales_Family_XIII	ILD	0.040220639
Peptostreptococcaceae	ILD	0.034474833
Clostridiales_Family_XI	ILD	0.028729028
Succinivibrionaceae	ILD	0.028729028
Thermaceae	ILD	0.028729028
Aerococcaceae	ILD	0.025856125
Paludibacteraceae	ILD	0.025856125
Bacteriovoracaceae	ILD	0.022983222
Mycobacteriaceae	ILD	0.022983222
Planococcaceae	ILD	0.020110319
Rhodocyclaceae	ILD	0.020110319
Rhodanobacteraceae	ILD	0.017237417
Rhizobiales_Incertae_Sedis	ILD	0.014364514
Lactobacillaceae	ILD	0.011491611
Enterococcaceae	ILD	0.008618708

Fimbriimonadaceae	ILD	0.008618708
Lentimicrobiaceae	ILD	0.008618708
Pirellulaceae	ILD	0.008618708
*Solibacteraceae_Subgroup_3_*	ILD	0.008618708
Barnesiellaceae	ILD	0.005745806
Chitinophagaceae	ILD	0.005745806
Propionibacteriaceae	ILD	0.005745806
Thermoactinomycetaceae	ILD	0.005745806
Coxiellaceae	ILD	0.002872903
Spirochaetaceae	ILD	0.002872903
Acetobacteraceae	ILD	0
Aeromonadaceae	ILD	0
Akkermansiaceae	ILD	0
Alteromonadaceae	ILD	0
Anaerolineaceae	ILD	0
Anaeroplasmataceae	ILD	0
Bacillales_Family_XII	ILD	0
Bacteroidales_F082	ILD	0
Betaproteobacteriales_TRA3_20	ILD	0
Blastocatellaceae	ILD	0
Candidatus_Saccharibacteria_bacterium_UB2523	ILD	0
Cardiobacteriaceae	ILD	0
Cellvibrionaceae	ILD	0
Chlamydiales_cvE6	ILD	0
Chromobacteriaceae	ILD	0
Chthoniobacteraceae	ILD	0
Clostridiales_vadinBB60_group	ILD	0
Coriobacteriales_Incertae_Sedis	ILD	0
Dermabacteraceae	ILD	0
Desulfovibrionaceae	ILD	0
Devosiaceae	ILD	0

Eggerthellaceae	ILD	0
Geminicoccaceae	ILD	0
Gemmataceae	ILD	0
Geodermatophilaceae	ILD	0
Halomonadaceae	ILD	0
Hydrogenophilaceae	ILD	0
Hymenobacteraceae	ILD	0
Ilumatobacteraceae	ILD	0
Intrasporangiaceae	ILD	0
Kaistiaceae	ILD	0
Legionellaceae	ILD	0
Leuconostocaceae	ILD	0
Listeriaceae	ILD	0
Marinifilaceae	ILD	0
Microbacteriaceae	ILD	0
Muribaculaceae	ILD	0
Mycoplasmataceae	ILD	0
Nocardiaceae	ILD	0
Nocardioidaceae	ILD	0
Oligoflexales_0319_6G20	ILD	0
Opitutaceae	ILD	0
Paenibacillaceae	ILD	0
Parvibaculaceae	ILD	0
Peptococcaceae	ILD	0
Pseudonocardiaceae	ILD	0
Rhodobacteraceae	ILD	0
Rickettsiaceae	ILD	0
Rikenellaceae	ILD	0
Rubinisphaeraceae	ILD	0
Sneathiellaceae	ILD	0
Sphingobacteriales_NS11_12_marine_group	ILD	0

Streptomycetaceae	ILD	0
Synergistaceae	ILD	0
Thermomicrobiales_JG30_KF_CM45	ILD	0
Pasteurellaceae	Stable COPD	12.6062974
Reyranellaceae	Stable COPD	11.90038415
Prevotellaceae	Stable COPD	10.91703057
Burkholderiaceae	Stable COPD	10.0962012
Enterobacteriaceae	Stable COPD	9.613553534
Veillonellaceae	Stable COPD	5.407623863
Streptococcaceae	Stable COPD	3.798798306
Ruminococcaceae	Stable COPD	3.65104902
Pseudomonadaceae	Stable COPD	3.168401353
Neisseriaceae	Stable COPD	3.048560265
Xanthobacteraceae	Stable COPD	2.97796894
Lachnospiraceae	Stable COPD	2.60202909
Rhizobiaceae	Stable COPD	2.176839479
Porphyromonadaceae	Stable COPD	2.12266474
Moraxellaceae	Stable COPD	1.761499819
Weeksellaceae	Stable COPD	1.620317168
Simkaniaceae	Stable COPD	1.137669501
Flavobacteriaceae	Stable COPD	0.86679581
Fusobacteriaceae	Stable COPD	0.817546049
Bacteroidaceae	Stable COPD	0.689496667
Sphingobacteriaceae	Stable COPD	0.669796763
Sphingomonadaceae	Stable COPD	0.628755294
Chitinophagaceae	Stable COPD	0.525330794
Xanthomonadaceae	Stable COPD	0.525330794
Erysipelotrichaceae	Stable COPD	0.472797715
Micrococcaceae	Stable COPD	0.338181699
Azospirillaceae	Stable COPD	0.311915159
Carnobacteriaceae	Stable COPD	0.293856913

Leptotrichiaceae	Stable COPD	0.277440326
Christensenellaceae	Stable COPD	0.252815445
Staphylococcaceae	Stable COPD	0.239682175
Tannerellaceae	Stable COPD	0.221623929
Campylobacteraceae	Stable COPD	0.216698953
Bacillales_Family_XI	Stable COPD	0.200282365
Coriobacteriaceae	Stable COPD	0.196999048
Akkermansiaceae	Stable COPD	0.167449191
Mycobacteriaceae	Stable COPD	0.162524214
*Solibacteraceae_Subgroup_3_*	Stable COPD	0.162524214
Corynebacteriaceae	Stable COPD	0.160882556
Actinomycetaceae	Stable COPD	0.141182651
Bifidobacteriaceae	Stable COPD	0.12969104
Saccharimonadaceae	Stable COPD	0.123124405
Atopobiaceae	Stable COPD	0.119841087
Lactobacillaceae	Stable COPD	0.118199429
Hyphomicrobiaceae	Stable COPD	0.113274453
Rikenellaceae	Stable COPD	0.109991135
Succinivibrionaceae	Stable COPD	0.105066159
Acidaminococcaceae	Stable COPD	0.100141183
Rhizobiales_Incertae_Sedis	Stable COPD	0.088649572
Beijerinckiaceae	Stable COPD	0.070591325
Peptostreptococcaceae	Stable COPD	0.068949667
Caulobacteraceae	Stable COPD	0.065666349
Spirochaetaceae	Stable COPD	0.065666349
Muribaculaceae	Stable COPD	0.060741373
Eggerthellaceae	Stable COPD	0.059099714
Clostridiales_Family_XIII	Stable COPD	0.049249762
Fimbriimonadaceae	Stable COPD	0.047608103
Clostridiales_Family_XI	Stable COPD	0.034474833
Legionellaceae	Stable COPD	0.032833175

Paludibacteraceae	Stable COPD	0.031191516
Aerococcaceae	Stable COPD	0.029549857
Propionibacteriaceae	Stable COPD	0.029549857
Rhodocyclaceae	Stable COPD	0.027908198
Clostridiales_vadinBB60_group	Stable COPD	0.02626654
Bacillales_Family_XII	Stable COPD	0.024624881
Lentimicrobiaceae	Stable COPD	0.024624881
Paenibacillaceae	Stable COPD	0.022983222
Alteromonadaceae	Stable COPD	0.021341564
Rhodanobacteraceae	Stable COPD	0.021341564
Marinifilaceae	Stable COPD	0.019699905
Rhodobacteraceae	Stable COPD	0.019699905
Chlamydiales_cvE6	Stable COPD	0.018058246
Parvibaculaceae	Stable COPD	0.018058246
Intrasporangiaceae	Stable COPD	0.016416587
Aeromonadaceae	Stable COPD	0.011491611
Bacillaceae	Stable COPD	0.011491611
Barnesiellaceae	Stable COPD	0.011491611
Chromobacteriaceae	Stable COPD	0.011491611
Hymenobacteraceae	Stable COPD	0.011491611
Pirellulaceae	Stable COPD	0.011491611
Synergistaceae	Stable COPD	0.011491611
Coriobacteriales_Incertae_Sedis	Stable COPD	0.009849952
Devosiaceae	Stable COPD	0.009849952
Mycoplasmataceae	Stable COPD	0.009849952
Nocardiaceae	Stable COPD	0.009849952
Bacteriovoracaceae	Stable COPD	0.008208294
Microbacteriaceae	Stable COPD	0.008208294
Candidatus_Saccharibacteria_bacterium_UB2523	Stable COPD	0.006566635
Cellvibrionaceae	Stable COPD	0.006566635
Peptococcaceae	Stable COPD	0.006566635

Thermomicrobiales_JG30_KF_CM45	Stable COPD	0.006566635
Leuconostocaceae	Stable COPD	0.004924976
Sphingobacteriales_NS11_12_marine_group	Stable COPD	0.004924976
Streptomycetaceae	Stable COPD	0.004924976
Anaeroplasmataceae	Stable COPD	0.003283317
Betaproteobacteriales_TRA3_20	Stable COPD	0.003283317
Cardiobacteriaceae	Stable COPD	0.003283317
Halomonadaceae	Stable COPD	0.003283317
Ilumatobacteraceae	Stable COPD	0.003283317
Oligoflexales_0319_6G20	Stable COPD	0.003283317
Pseudonocardiaceae	Stable COPD	0.003283317
Rubinisphaeraceae	Stable COPD	0.003283317
Acetobacteraceae	Stable COPD	0.001641659
Anaerolineaceae	Stable COPD	0.001641659
Bacteroidales_F082	Stable COPD	0.001641659
Coxiellaceae	Stable COPD	0.001641659
Listeriaceae	Stable COPD	0.001641659
Nocardioidaceae	Stable COPD	0.001641659
Rickettsiaceae	Stable COPD	0.001641659
Sneathiellaceae	Stable COPD	0.001641659
Blastocatellaceae	Stable COPD	0
Chthoniobacteraceae	Stable COPD	0
Dermabacteraceae	Stable COPD	0
Desulfovibrionaceae	Stable COPD	0
Enterococcaceae	Stable COPD	0
Geminicoccaceae	Stable COPD	0
Gemmataceae	Stable COPD	0
Geodermatophilaceae	Stable COPD	0
Hydrogenophilaceae	Stable COPD	0
Kaistiaceae	Stable COPD	0
Opitutaceae	Stable COPD	0

Planococcaceae	Stable COPD	0
Thermaceae	Stable COPD	0
Thermoactinomycetaceae	Stable COPD	0
Prevotellaceae	Sarcoidosis	13.4250747
Burkholderiaceae	Sarcoidosis	12.09204781
Streptococcaceae	Sarcoidosis	11.33647437
Enterobacteriaceae	Sarcoidosis	10.14421972
Reyranellaceae	Sarcoidosis	8.624454148
Neisseriaceae	Sarcoidosis	5.616524937
Xanthobacteraceae	Sarcoidosis	5.240174672
Micrococcaceae	Sarcoidosis	4.677085727
Veillonellaceae	Sarcoidosis	4.521948977
Rhizobiaceae	Sarcoidosis	3.562399448
Pasteurellaceae	Sarcoidosis	3.160193059
Carnobacteriaceae	Sarcoidosis	2.378763503
Porphyromonadaceae	Sarcoidosis	1.669156516
Xanthomonadaceae	Sarcoidosis	1.335899793
Fusobacteriaceae	Sarcoidosis	1.289933349
Sphingomonadaceae	Sarcoidosis	1.028499196
Weeksellaceae	Sarcoidosis	0.985405654
Pseudomonadaceae	Sarcoidosis	0.942312112
Sphingobacteriaceae	Sarcoidosis	0.864743737
Staphylococcaceae	Sarcoidosis	0.680877959
Flavobacteriaceae	Sarcoidosis	0.675132154
Leptotrichiaceae	Sarcoidosis	0.597563778
Simkaniaceae	Sarcoidosis	0.580326362
Lachnospiraceae	Sarcoidosis	0.560216042
Bacillales_Family_XI	Sarcoidosis	0.425189612
Actinomycetaceae	Sarcoidosis	0.367731556
Ruminococcaceae	Sarcoidosis	0.304527695
Corynebacteriaceae	Sarcoidosis	0.249942542

Azospirillaceae	Sarcoidosis	0.201103195
Campylobacteraceae	Sarcoidosis	0.152263847
Caulobacteraceae	Sarcoidosis	0.146518042
Rhodocyclaceae	Sarcoidosis	0.140772236
Erysipelotrichaceae	Sarcoidosis	0.137899333
Saccharimonadaceae	Sarcoidosis	0.112043208
Mycobacteriaceae	Sarcoidosis	0.100551597
Chitinophagaceae	Sarcoidosis	0.097678695
Hyphomicrobiaceae	Sarcoidosis	0.097678695
Clostridiales_Family_XIII	Sarcoidosis	0.089059986
Peptostreptococcaceae	Sarcoidosis	0.077568375
Spirochaetaceae	Sarcoidosis	0.077568375
Bacteriovoracaceae	Sarcoidosis	0.07182257
Moraxellaceae	Sarcoidosis	0.07182257
Rhizobiales_Incertae_Sedis	Sarcoidosis	0.068949667
Beijerinckiaceae	Sarcoidosis	0.054585153
Atopobiaceae	Sarcoidosis	0.048839347
Mycoplasmataceae	Sarcoidosis	0.045966444
*Solibacteraceae_Subgroup_3_*	Sarcoidosis	0.045966444
Bifidobacteriaceae	Sarcoidosis	0.043093542
Christensenellaceae	Sarcoidosis	0.043093542
Rhodanobacteraceae	Sarcoidosis	0.043093542
Aerococcaceae	Sarcoidosis	0.037347736
Clostridiales_Family_XI	Sarcoidosis	0.037347736
Bacillaceae	Sarcoidosis	0.028729028
Coriobacteriaceae	Sarcoidosis	0.028729028
Microbacteriaceae	Sarcoidosis	0.025856125
Propionibacteriaceae	Sarcoidosis	0.020110319
Tannerellaceae	Sarcoidosis	0.020110319
Aeromonadaceae	Sarcoidosis	0.017237417
Blastocatellaceae	Sarcoidosis	0.017237417

Lentimicrobiaceae	Sarcoidosis	0.017237417
Thermaceae	Sarcoidosis	0.017237417
Nocardiaceae	Sarcoidosis	0.014364514
Paludibacteraceae	Sarcoidosis	0.014364514
Clostridiales_vadinBB60_group	Sarcoidosis	0.011491611
Desulfovibrionaceae	Sarcoidosis	0.011491611
Devosiaceae	Sarcoidosis	0.011491611
Fimbriimonadaceae	Sarcoidosis	0.011491611
Geminicoccaceae	Sarcoidosis	0.011491611
Lactobacillaceae	Sarcoidosis	0.011491611
Acetobacteraceae	Sarcoidosis	0.008618708
Marinifilaceae	Sarcoidosis	0.008618708
Parvibaculaceae	Sarcoidosis	0.008618708
Rikenellaceae	Sarcoidosis	0.008618708
Bacillales_Family_XII	Sarcoidosis	0.005745806
Candidatus_Saccharibacteria_bacterium_UB2523	Sarcoidosis	0.005745806
Kaistiaceae	Sarcoidosis	0.005745806
Planococcaceae	Sarcoidosis	0.005745806
Rubinisphaeraceae	Sarcoidosis	0.005745806
Synergistaceae	Sarcoidosis	0.005745806
Cardiobacteriaceae	Sarcoidosis	0.002872903
Chlamydiales_cvE6	Sarcoidosis	0.002872903
Gemmataceae	Sarcoidosis	0.002872903
Halomonadaceae	Sarcoidosis	0.002872903
Pirellulaceae	Sarcoidosis	0.002872903
Sneathiellaceae	Sarcoidosis	0.002872903
Acidaminococcaceae	Sarcoidosis	0
Akkermansiaceae	Sarcoidosis	0
Alteromonadaceae	Sarcoidosis	0
Anaerolineaceae	Sarcoidosis	0
Anaeroplasmataceae	Sarcoidosis	0

Bacteroidaceae	Sarcoidosis	0
Bacteroidales_F082	Sarcoidosis	0
Barnesiellaceae	Sarcoidosis	0
Betaproteobacteriales_TRA3_20	Sarcoidosis	0
Cellvibrionaceae	Sarcoidosis	0
Chromobacteriaceae	Sarcoidosis	0
Chthoniobacteraceae	Sarcoidosis	0
Coriobacteriales_Incertae_Sedis	Sarcoidosis	0
Coxiellaceae	Sarcoidosis	0
Dermabacteraceae	Sarcoidosis	0
Eggerthellaceae	Sarcoidosis	0
Enterococcaceae	Sarcoidosis	0
Geodermatophilaceae	Sarcoidosis	0
Hydrogenophilaceae	Sarcoidosis	0
Hymenobacteraceae	Sarcoidosis	0
Ilumatobacteraceae	Sarcoidosis	0
Intrasporangiaceae	Sarcoidosis	0
Legionellaceae	Sarcoidosis	0
Leuconostocaceae	Sarcoidosis	0
Listeriaceae	Sarcoidosis	0
Muribaculaceae	Sarcoidosis	0
Nocardioidaceae	Sarcoidosis	0
Oligoflexales_0319_6G20	Sarcoidosis	0
Opitutaceae	Sarcoidosis	0
Paenibacillaceae	Sarcoidosis	0
Peptococcaceae	Sarcoidosis	0
Pseudonocardiaceae	Sarcoidosis	0
Rhodobacteraceae	Sarcoidosis	0
Rickettsiaceae	Sarcoidosis	0
Sphingobacteriales_NS11_12_marine_group	Sarcoidosis	0
Streptomycetaceae	Sarcoidosis	0

Succinivibrionaceae	Sarcoidosis	0
Thermoactinomycetaceae	Sarcoidosis	0
Thermomicrobiales_JG30_KF_CM45	Sarcoidosis	0

Supplementary Table S6: List of differentially abundant taxa in the disease group determined by LEfSe analysis.

**Taxa**	**Disease**	**LDA value**	**p-value**
Bacteria.Firmicutes.Bacilli.Lactobacillales.Streptococcaceae.Streptococcus.OTUID_926	Exacerbated COPD	4.572829633	0.0323923
Bacteria.Firmicutes.Clostridia.Clostridiales.Ruminococcaceae.Faecalibacterium	Exacerbated COPD	3.626306434	0.04071854
Bacteria.Firmicutes.Clostridia.Clostridiales.Lachnospiraceae.Coprococcus	Exacerbated COPD	3.159305516	0.013919972
Bacteria.Proteobacteria.Gammaproteobacteria.Pasteurellales.Pasteurellaceae.Haemophilus.OTUID_375	ILD	4.4636194	0.049337409
Bacteria.Proteobacteria.Gammaproteobacteria.Xanthomonadales.Xanthomonadaceae.Stenotrophomonas.OTUID_166	ILD	3.751730583	0.018698125
Bacteria.Proteobacteria.Gammaproteobacteria.Pasteurellales.Pasteurellaceae.Haemophilus.OTUID_354	ILD	3.687481704	0.029850028
Bacteria.Proteobacteria.Gammaproteobacteria.Enterobacteriales.Enterobacteriaceae.OTUID_238	ILD	3.176452782	0.048171506
Bacteria.Proteobacteria.Gammaproteobacteria.Pasteurellales.Pasteurellaceae.Actinobacillus	Stable COPD	4.30726047	0.000387691
Bacteria.Proteobacteria.Gammaproteobacteria.Pasteurellales.Pasteurellaceae.Actinobacillus.OTUID_381	Stable COPD	4.14427881	0.00465136
Bacteria.Proteobacteria.Gammaproteobacteria.Betaproteobacteriales.Burkholderiaceae.Massilia	Stable COPD	3.78714392	0.036232632
Bacteria.Proteobacteria.Alphaproteobacteria.Reyranellales.Reyranellaceae.Reyranella.OTUID_59	Stable COPD	3.707268185	6.82336E-05
Bacteria.Chlamydiae	Stable COPD	3.661756478	0.014600486
Bacteria.Chlamydiae.Chlamydiae.Chlamydiales	Stable COPD	3.660149387	0.014600486
Bacteria.Chlamydiae.Chlamydiae	Stable COPD	3.658384673	0.014600486
Bacteria.Chlamydiae.Chlamydiae.Chlamydiales.Simkaniaceae	Stable COPD	3.656901461	0.018910204
Bacteria.Chlamydiae.Chlamydiae.Chlamydiales.Simkaniaceae.OTUID_48	Stable COPD	3.6551978	0.018910204
Bacteria.Firmicutes.Negativicutes.Selenomonadales.Veillonellaceae.Megasphaera	Stable COPD	3.476029765	0.040711691
Bacteria.Firmicutes.Negativicutes.Selenomonadales.Veillonellaceae.Dialister	Stable COPD	3.474076339	0.009051633
Bacteria.Proteobacteria.Gammaproteobacteria.Pasteurellales.Pasteurellaceae.Actinobacillus.OTUID_380	Stable COPD	3.459572182	0.038652311
Bacteria.Proteobacteria.Gammaproteobacteria.Enterobacteriales.Enterobacteriaceae.Enterobacter.OTUID_198	Stable COPD	3.454489	0.030698381
Bacteria.Bacteroidetes.Bacteroidia.Chitinophagales.Chitinophagaceae	Stable COPD	3.41185366	0.000176602
Bacteria.Bacteroidetes.Bacteroidia.Chitinophagales	Stable COPD	3.411519919	0.000176602
Bacteria.Proteobacteria.Gammaproteobacteria.Pasteurellales.Pasteurellaceae.Aggregatibacter	Stable COPD	3.38711086	0.028447531

Bacteria.Proteobacteria.Gammaproteobacteria.Betaproteobacteriales.Burkholderiaceae.Achromobacter.OTUID_444	Stable COPD	3.331072602	0.049869671
Bacteria.Bacteroidetes.Bacteroidia.Chitinophagales.Chitinophagaceae.Chitinophaga	Stable COPD	3.326248984	0.0006531
Bacteria.Firmicutes.Negativicutes.Selenomonadales.Veillonellaceae.Dialister.OTUID_1103	Stable COPD	3.311041847	0.046541121
Bacteria.Bacteroidetes.Bacteroidia.Flavobacteriales.Weeksellaceae.Elizabethkingia	Stable COPD	3.300208994	0.022848053
Bacteria.Bacteroidetes.Bacteroidia.Flavobacteriales.Weeksellaceae.Elizabethkingia.OTUID_2056	Stable COPD	3.296872222	0.022848053
Bacteria.Proteobacteria.Alphaproteobacteria.Rhizobiales.Rhizobiaceae.Mesorhizobium.OTUID_143	Stable COPD	3.150616596	0.000209963
Bacteria.Bacteroidetes.Bacteroidia.Chitinophagales.Chitinophagaceae.Chitinophaga.OTUID_1629	Stable COPD	3.129029406	0.015070339
Bacteria.Bacteroidetes.Bacteroidia.Bacteroidales.Prevotellaceae.Prevotella.OTUID_1881	Stable COPD	3.124220686	0.041045906
Bacteria.Proteobacteria.Alphaproteobacteria.Azospirillales.Azospirillaceae	Stable COPD	3.08510999	0.026530685
Bacteria.Proteobacteria.Alphaproteobacteria.Azospirillales	Stable COPD	3.084630659	0.026530685
Bacteria.Proteobacteria.Alphaproteobacteria.Azospirillales.Azospirillaceae.Azospirillum	Stable COPD	3.082379694	0.026530685
Bacteria.Proteobacteria.Alphaproteobacteria.Azospirillales.Azospirillaceae.Azospirillum.OTUID_60	Stable COPD	3.079957631	0.026530685
Bacteria.Proteobacteria.Gammaproteobacteria.Pasteurellales.Pasteurellaceae.Haemophilus.OTUID_335	Stable COPD	3.046929561	0.030698381
Bacteria.Firmicutes.Erysipelotrichia.Erysipelotrichales.Erysipelotrichaceae.Holdemanella	Stable COPD	3.008763238	0.022407549
Bacteria.Actinobacteria.Actinobacteria.Corynebacteriales	Sarcoidosis	3.204804954	0.010923783
Bacteria.Actinobacteria.Actinobacteria.Corynebacteriales.Corynebacteriaceae.Corynebacterium	Sarcoidosis	3.069109508	0.032611055
Bacteria.Proteobacteria.Gammaproteobacteria.Betaproteobacteriales.Neisseriaceae.Neisseria.OTUID_476	Sarcoidosis	3.032090323	0.029850028

Supplementary Table S7: List of ASVs and their taxonomic classification obtained through core microbiome analysis for disease group.

ASV ID	Disease	Kingdom	Phylum	Class	Order	Family	Genus	Species
d44b60b26ebc01728a51b8a1cd5d9ccc	Exacerbated COPD	Bacteria	Proteobacteria	Alphaproteobacteria	*Reyranellales*	*Reyranellaceae*	*Reyranella*	
e56516a9aabcee32a7426bd3894861a2	Exacerbated COPD	Bacteria	Proteobacteria	Alphaproteobacteria	*Sphingomonadales*	*Sphingomonadaceae*	*Sphingomonas*	
21b372faee1e8cf4fff63fb919ce52b2	Exacerbated COPD	Bacteria	Proteobacteria	Alphaproteobacteria	*Rhizobiales*	*Xanthobacteraceae*		
bbf2fc899ad681cdaf0b9eb914c37cdd	Exacerbated COPD	Bacteria	Proteobacteria	Alphaproteobacteria	*Rhizobiales*	*Xanthobacteraceae*		
ca0285f0e074a69bf90f7fb6763ca7cc	Exacerbated COPD	Bacteria	Proteobacteria	Alphaproteobacteria	*Rhizobiales*	*Rhizobiaceae*	*Ochrobactrum*	
fc71ae7eda4a4430ce3474b70abc6a68	Exacerbated COPD	Bacteria	Proteobacteria	Alphaproteobacteria	*Rhizobiales*	*Rhizobiaceae*	*Mesorhizobium*	
c3dd369e13ccb4d1b19bc1aac91917ca	Exacerbated COPD	Bacteria	Proteobacteria	Gammaproteobacteria	*Xanthomonadales*	*Xanthomonadaceae*	*Stenotrophomonas*	
c6f0efea3a269137720ca341ec873169	Exacerbated COPD	Bacteria	Proteobacteria	Gammaproteobacteria	*Enterobacteriales*	*Enterobacteriaceae*	*Escherichia_Shigella*	
584a15a8b8273021c7e3e7d07d3bc48d	Exacerbated COPD	Bacteria	Proteobacteria	Gammaproteobacteria	*Betaproteobacteriales*	*Burkholderiaceae*	*Ralstonia*	
7f1495320cf00e9f81e455ea44145d2c	Exacerbated COPD	Bacteria	Proteobacteria	Gammaproteobacteria	*Betaproteobacteriales*	*Burkholderiaceae*	*Achromobacter*	
616cd8134b106d6ed8e9cd6edce9a82a	Exacerbated COPD	Bacteria	Proteobacteria	Gammaproteobacteria	*Pseudomonadales*	*Pseudomonadaceae*	*Pseudomonas*	*Pseudomonas_aeruginosa*
b8a03689f1c69c6623c731cce70d1f4a	Exacerbated COPD	Bacteria	Fusobacteria	Fusobacteriia	*Fusobacteriales*	*Fusobacteriaceae*	*Fusobacterium*	
76ba84783044dc63a25709724d12740f	Exacerbated COPD	Bacteria	Firmicutes	Bacilli	*Lactobacillales*	*Streptococcaceae*	*Streptococcus*	
9adcea5ae4bfb4764d7ac5025de92c6d	Exacerbated COPD	Bacteria	Firmicutes	Bacilli	*Lactobacillales*	*Streptococcaceae*	*Streptococcus*	

9160e6eb439750e23c5b6b6976e8f999	Exacerbated COPD	Bacteria	Firmicutes	Erysipelotrichia	*Erysipelotrichales*	*Erysipelotrichaceae*	*Catenibacterium*
642c42a9c0ccb1cfcabd86e2205edbf8	Exacerbated COPD	Bacteria	Firmicutes	Bacilli	*Bacillales*	*Bacillales_Family_XI*	*Gemella*
b15c7ffc9025f0458b58978326cd99b9	Exacerbated COPD	Bacteria	Firmicutes	Bacilli	*Lactobacillales*	*Carnobacteriaceae*	*Granulicatella*
78d779cecdb01d5dd1e32a4e98509039	Exacerbated COPD	Bacteria	Firmicutes	Negativicutes	*Selenomonadales*	*Veillonellaceae*	*Dialister*
12c9f21b276ff04fb4caf797cbcbf68e	Exacerbated COPD	Bacteria	Firmicutes	Clostridia	*Clostridiales*	*Christensenellaceae*	*Christensenellaceae _R_7_group*
477bbdf91d68dceea38040dd249ba3e8	Exacerbated COPD	Bacteria	Firmicutes	Clostridia	*Clostridiales*	*Ruminococcaceae*	*Subdoligranulum*
72930a2e533f2c174341b760550850c9	Exacerbated COPD	Bacteria	Bacteroidetes	Bacteroidia	*Bacteroidales*	*Prevotellaceae*	*Prevotella*
ab7c6ac58527511c2256ec4eded035f8	Exacerbated COPD	Bacteria	Bacteroidetes	Bacteroidia	*Flavobacteriales*	*Weeksellaceae*	*Chryseobacterium*
1c0b01a1271927fd61457bee46e6d4be	Stable COPD	Bacteria	Epsilonbacteraeota	Campylobacteria	*Campylobacterales*	*Campylobacteraceae*	*Campylobacter*
43c574f67b0c843c24be7ff59058603b	Stable COPD	Bacteria	Chlamydiae	Chlamydiae	*Chlamydiales*	*Simkaniaceae*	
d44b60b26ebc01728a51b8a1cd5d9ccc	Stable COPD	Bacteria	Proteobacteria	Alphaproteobacteria	*Reyranellales*	*Reyranellaceae*	*Reyranella*
b8e86a79aa5a318d12e80eece549b4b4	Stable COPD	Bacteria	Proteobacteria	Alphaproteobacteria	*Reyranellales*	*Reyranellaceae*	*Reyranella*
bae07d3bb70f0fa015ec481fc82a61d3	Stable COPD	Bacteria	Proteobacteria	Alphaproteobacteria	*Azospirillales*	*Azospirillaceae*	*Azospirillum*
e56516a9aabcee32a7426bd3894861a2	Stable COPD	Bacteria	Proteobacteria	Alphaproteobacteria	*Sphingomonadales*	*Sphingomonadaceae*	*Sphingomonas*
21b372faee1e8cf4fff63fb919ce52b2	Stable COPD	Bacteria	Proteobacteria	Alphaproteobacteria	*Rhizobiales*	*Xanthobacteraceae*	
bbf2fc899ad681cdaf0b9eb914c37cdd	Stable COPD	Bacteria	Proteobacteria	Alphaproteobacteria	*Rhizobiales*	*Xanthobacteraceae*	
ca0285f0e074a69bf90f7fb6763ca7cc	Stable COPD	Bacteria	Proteobacteria	Alphaproteobacteria	*Rhizobiales*	*Rhizobiaceae*	*Ochrobactrum*
fc71ae7eda4a4430ce3474b70abc6a68	Stable COPD	Bacteria	Proteobacteria	Alphaproteobacteria	*Rhizobiales*	*Rhizobiaceae*	*Mesorhizobium*
b283956b5c8dca5e6122dcd0ede1a45e	Stable COPD	Bacteria	Proteobacteria	Alphaproteobacteria	*Rhizobiales*	*Rhizobiaceae*	*Mesorhizobium*

0a4415ddaa4e2748976f0087a87d0f86	Stable COPD	Bacteria	Proteobacteria	Alphaproteobacteria	*Rhizobiales*	*Hyphomicrobiaceae*	*Hyphomicrobium*	
5c90f1226258dccd1314b3da5fb50686	Stable COPD	Bacteria	Proteobacteria	Gammaproteobacteria	*Xanthomonadales*	*Xanthomonadaceae*	*Pseudoxanthomonas*	
e648ca13dc100dda271c75f8fea94de9	Stable COPD	Bacteria	Proteobacteria	Gammaproteobacteria	*Xanthomonadales*	*Xanthomonadaceae*	*Stenotrophomonas*	
c6f0efea3a269137720ca341ec873169	Stable COPD	Bacteria	Proteobacteria	Gammaproteobacteria	*Enterobacteriales*	*Enterobacteriaceae*	*Escherichia_Shigella*	
ed33d85165cf28fced2062b5c09b906c	Stable COPD	Bacteria	Proteobacteria	Gammaproteobacteria	*Pasteurellales*	*Pasteurellaceae*	*Haemophilus*	
a0df70628cd44941e49b90ef475435a6	Stable COPD	Bacteria	Proteobacteria	Gammaproteobacteria	*Pasteurellales*	*Pasteurellaceae*	*Actinobacillus*	
584a15a8b8273021c7e3e7d07d3bc48d	Stable COPD	Bacteria	Proteobacteria	Gammaproteobacteria	*Betaproteobacteriales*	*Burkholderiaceae*	*Ralstonia*	
62e3189638c1d1ca7e69cbdbf869d5d0	Stable COPD	Bacteria	Proteobacteria	Gammaproteobacteria	*Betaproteobacteriales*	*Burkholderiaceae*	*Massilia*	
7e6f3182e920f78b045bd815eda5b9d9	Stable COPD	Bacteria	Proteobacteria	Gammaproteobacteria	*Betaproteobacteriales*	*Burkholderiaceae*	*Achromobacter*	
7f1495320cf00e9f81e455ea44145d2c	Stable COPD	Bacteria	Proteobacteria	Gammaproteobacteria	*Betaproteobacteriales*	*Burkholderiaceae*	*Achromobacter*	
616cd8134b106d6ed8e9cd6edce9a82a	Stable COPD	Bacteria	Proteobacteria	Gammaproteobacteria	*Pseudomonadales*	*Pseudomonadaceae*	*Pseudomonas*	*Pseudomonas_aeruginosa*
96c77409f836c79532629b787bdf4f7f	Stable COPD	Bacteria	Proteobacteria	Gammaproteobacteria	*Pseudomonadales*	*Pseudomonadaceae*	*Pseudomonas*	*Pseudomonas_stutzeri*
3e93a180549b96db8995ed7cc6d6eaa8	Stable COPD	Bacteria	Proteobacteria	Gammaproteobacteria				
75a7f524e7dd1a59e9fac8a248da9e40	Stable COPD	Bacteria	Actinobacteria	Actinobacteria	*Corynebacteriales*	*Mycobacteriaceae*	*Mycobacterium*	
dc5b26848cac5ada34a8293bcecc7408	Stable COPD	Bacteria	Acidobacteria	Acidobacteriia	*Solibacterales*	*Solibacteraceae_Subgroup_3_*	*Paludibaculum*	
b8a03689f1c69c6623c731cce70d1f4a	Stable COPD	Bacteria	Fusobacteria	Fusobacteriia	*Fusobacteriales*	*Fusobacteriaceae*	*Fusobacterium*	
76ba84783044dc63a25709724d12740f	Stable COPD	Bacteria	Firmicutes	Bacilli	*Lactobacillales*	*Streptococcaceae*	*Streptococcus*	
642c42a9c0ccb1cfcabd86e2205edbf8	Stable COPD	Bacteria	Firmicutes	Bacilli	*Bacillales*	*Bacillales_Family_XI*	*Gemella*	
b15c7ffc9025f0458b58978326cd99b9	Stable COPD	Bacteria	Firmicutes	Bacilli	*Lactobacillales*	*Carnobacteriaceae*	*Granulicatella*	
78d779cecdb01d5dd1e32a4e98509039	Stable COPD	Bacteria	Firmicutes	Negativicutes	*Selenomonadales*	*Veillonellaceae*	*Dialister*	
e7c5690166d106cd3e6f50e7a7f36562	Stable COPD	Bacteria	Firmicutes	Negativicutes	*Selenomonadales*	*Veillonellaceae*	*Veillonella*	

0362fa463ae8c79c2e4e013b873166ac	Stable COPD	Bacteria	Bacteroidetes	Bacteroidia	*Chitinophagales*	*Chitinophagaceae*	*Chitinophaga*	
72930a2e533f2c174341b760550850c9	Stable COPD	Bacteria	Bacteroidetes	Bacteroidia	*Bacteroidales*	*Prevotellaceae*	*Prevotella*	
dec59104e90212646e1ab72f2db4f326	Stable COPD	Bacteria	Bacteroidetes	Bacteroidia	*Flavobacteriales*	*Weeksellaceae*	*Elizabethkingia*	
ab7c6ac58527511c2256ec4eded035f8	Stable COPD	Bacteria	Bacteroidetes	Bacteroidia	*Flavobacteriales*	*Weeksellaceae*	*Chryseobacterium*	
de287dd37785fcd58644cfeeabcd45f4	Stable COPD	Bacteria	Bacteroidetes	Bacteroidia	*Sphingobacteriales*	*Sphingobacteriaceae*	*Sphingobacterium*	*Sphingobacterium_spiritivorum*
0d335e6d528bb27c55a31539feb8517f	Stable COPD	Bacteria	Firmicutes	Clostridia	*Clostridiales*	*Lachnospiraceae*	*Agathobacter*	
1c0b01a1271927fd61457bee46e6d4be	Sarcoidosis	Bacteria	Epsilonbacteraeota	Campylobacteria	*Campylobacterales*	*Campylobacteraceae*	*Campylobacter*	
43c574f67b0c843c24be7ff59058603b	Sarcoidosis	Bacteria	Chlamydiae	Chlamydiae	*Chlamydiales*	*Simkaniaceae*		
d44b60b26ebc01728a51b8a1cd5d9ccc	Sarcoidosis	Bacteria	Proteobacteria	Alphaproteobacteria	*Reyranellales*	*Reyranellaceae*	*Reyranella*	
bae07d3bb70f0fa015ec481fc82a61d3	Sarcoidosis	Bacteria	Proteobacteria	Alphaproteobacteria	*Azospirillales*	*Azospirillaceae*	*Azospirillum*	
e56516a9aabcee32a7426bd3894861a2	Sarcoidosis	Bacteria	Proteobacteria	Alphaproteobacteria	*Sphingomonadales*	*Sphingomonadaceae*	*Sphingomonas*	
21b372faee1e8cf4fff63fb919ce52b2	Sarcoidosis	Bacteria	Proteobacteria	Alphaproteobacteria	*Rhizobiales*	*Xanthobacteraceae*		
34b943abb7623f70ea626ede05f44458	Sarcoidosis	Bacteria	Proteobacteria	Alphaproteobacteria	*Rhizobiales*	*Xanthobacteraceae*	*Bradyrhizobium*	
bbf2fc899ad681cdaf0b9eb914c37cdd	Sarcoidosis	Bacteria	Proteobacteria	Alphaproteobacteria	*Rhizobiales*	*Xanthobacteraceae*		
047da6a283557bfaf7dca1047d4cef27	Sarcoidosis	Bacteria	Proteobacteria	Alphaproteobacteria	*Rhizobiales*	*Rhizobiales_Incertae_Sedis*	*Nordella*	
ca0285f0e074a69bf90f7fb6763ca7cc	Sarcoidosis	Bacteria	Proteobacteria	Alphaproteobacteria	*Rhizobiales*	*Rhizobiaceae*	*Ochrobactrum*	
fc71ae7eda4a4430ce3474b70abc6a68	Sarcoidosis	Bacteria	Proteobacteria	Alphaproteobacteria	*Rhizobiales*	*Rhizobiaceae*	*Mesorhizobium*	
c3dd369e13ccb4d1b19bc1aac91917ca	Sarcoidosis	Bacteria	Proteobacteria	Gammaproteobacteria	*Xanthomonadales*	*Xanthomonadaceae*	*Stenotrophomonas*	
a4612e820ea7a0cc568833ed8ad0b4e6	Sarcoidosis	Bacteria	Proteobacteria	Gammaproteobacteria	*Xanthomonadales*	*Xanthomonadaceae*	*Vulcaniibacterium*	
ed33d85165cf28fced2062b5c09b906c	Sarcoidosis	Bacteria	Proteobacteria	Gammaproteobacteria	*Pasteurellales*	*Pasteurellaceae*	*Haemophilus*	
584a15a8b8273021c7e3e7d07d3bc48d	Sarcoidosis	Bacteria	Proteobacteria	Gammaproteobacteria	*Betaproteobacteriales*	*Burkholderiaceae*	*Ralstonia*	
62e3189638c1d1ca7e69cbdbf869d5d0	Sarcoidosis	Bacteria	Proteobacteria	Gammaproteobacteria	*Betaproteobacteriales*	*Burkholderiaceae*	*Massilia*	
7e6f3182e920f78b045bd815eda5b9d9	Sarcoidosis	Bacteria	Proteobacteria	Gammaproteobacteria	*Betaproteobacteriales*	*Burkholderiaceae*	*Achromobacter*	
7f1495320cf00e9f81e455ea44145d2c	Sarcoidosis	Bacteria	Proteobacteria	Gammaproteobacteria	*Betaproteobacteriales*	*Burkholderiaceae*	*Achromobacter*	

0a9e2ee24e414199dc0e7e90bdd3c33f	Sarcoidosis	Bacteria	Proteobacteria	Gammaproteobacteria	*Betaproteobacteriales*	*Rhodocyclaceae*	*Methyloversatilis*	
c6eb5c0b8347faff7c918df42bbf1f29	Sarcoidosis	Bacteria	Proteobacteria	Gammaproteobacteria	*Betaproteobacteriales*	*Burkholderiaceae*	*Variovorax*	
616cd8134b106d6ed8e9cd6edce9a82a	Sarcoidosis	Bacteria	Proteobacteria	Gammaproteobacteria	*Pseudomonadales*	*Pseudomonadaceae*	*Pseudomonas*	*Pseudomonas_aeruginosa*
d3b4fcc82367e8b333215f14ab29f9fe	Sarcoidosis	Bacteria	Firmicutes	Bacilli	*Lactobacillales*	*Streptococcaceae*	*Streptococcus*	*Streptococcus_salivarius_subsp_thermophilus*
76ba84783044dc63a25709724d12740f	Sarcoidosis	Bacteria	Firmicutes	Bacilli	*Lactobacillales*	*Streptococcaceae*	*Streptococcus*	
7a17b0aeeb69be8bb0aa127caf00b5d7	Sarcoidosis	Bacteria	Firmicutes	Bacilli	*Lactobacillales*	*Streptococcaceae*	*Streptococcus*	
642c42a9c0ccb1cfcabd86e2205edbf8	Sarcoidosis	Bacteria	Firmicutes	Bacilli	*Bacillales*	*Bacillales_Family_XI*	*Gemella*	
ea23c38595086f7793f76cf307817dd2	Sarcoidosis	Bacteria	Firmicutes	Bacilli	*Bacillales*	*Staphylococcaceae*	*Staphylococcus*	
b15c7ffc9025f0458b58978326cd99b9	Sarcoidosis	Bacteria	Firmicutes	Bacilli	*Lactobacillales*	*Carnobacteriaceae*	*Granulicatella*	
e7c5690166d106cd3e6f50e7a7f36562	Sarcoidosis	Bacteria	Firmicutes	Negativicutes	*Selenomonadales*	*Veillonellaceae*	*Veillonella*	
b5022e5e3ff38bbeda64958306fe94dc	Sarcoidosis	Bacteria	Firmicutes	Negativicutes	*Selenomonadales*	*Veillonellaceae*	*Veillonella*	
126eb96d7899f09b820a34aa763e01c3	Sarcoidosis	Bacteria	Firmicutes	Negativicutes	*Selenomonadales*	*Veillonellaceae*	*Veillonella*	
72930a2e533f2c174341b760550850c9	Sarcoidosis	Bacteria	Bacteroidetes	Bacteroidia	*Bacteroidales*	*Prevotellaceae*	*Prevotella*	
286131addec0b1e8574145afaafc7f7c	Sarcoidosis	Bacteria	Bacteroidetes	Bacteroidia	*Bacteroidales*	*Prevotellaceae*	*Prevotella*	
dec59104e90212646e1ab72f2db4f326	Sarcoidosis	Bacteria	Bacteroidetes	Bacteroidia	*Flavobacteriales*	*Weeksellaceae*	*Elizabethkingia*	
ab7c6ac58527511c2256ec4eded035f8	Sarcoidosis	Bacteria	Bacteroidetes	Bacteroidia	*Flavobacteriales*	*Weeksellaceae*	*Chryseobacterium*	
de287dd37785fcd58644cfeeabcd45f4	Sarcoidosis	Bacteria	Bacteroidetes	Bacteroidia	*Sphingobacteriales*	*Sphingobacteriaceae*	*Sphingobacterium*	*Sphingobacterium_spiritivorum*
43c574f67b0c843c24be7ff59058603b	ILD	Bacteria	Chlamydiae	Chlamydiae	*Chlamydiales*	*Simkaniaceae*		
d44b60b26ebc01728a51b8a1cd5d9ccc	ILD	Bacteria	Proteobacteria	Alphaproteobacteria	*Reyranellales*	*Reyranellaceae*	*Reyranella*	
bae07d3bb70f0fa015ec481fc82a61d3	ILD	Bacteria	Proteobacteria	Alphaproteobacteria	*Azospirillales*	*Azospirillaceae*	*Azospirillum*	
21b372faee1e8cf4fff63fb919ce52b2	ILD	Bacteria	Proteobacteria	Alphaproteobacteria	*Rhizobiales*	*Xanthobacteraceae*		
bbf2fc899ad681cdaf0b9eb914c37cdd	ILD	Bacteria	Proteobacteria	Alphaproteobacteria	*Rhizobiales*	*Xanthobacteraceae*		

ca0285f0e074a69bf90f7fb6763ca7cc	ILD	Bacteria	Proteobacteria	Alphaproteobacteria	*Rhizobiales*	*Rhizobiaceae*	*Ochrobactrum*	
fc71ae7eda4a4430ce3474b70abc6a68	ILD	Bacteria	Proteobacteria	Alphaproteobacteria	*Rhizobiales*	*Rhizobiaceae*	*Mesorhizobium*	
c3dd369e13ccb4d1b19bc1aac91917ca	ILD	Bacteria	Proteobacteria	Gammaproteobacteria	*Xanthomonadales*	*Xanthomonadaceae*	*Stenotrophomonas*	
0cf4a52b4562a6b2fb3404e74a2db527	ILD	Bacteria	Proteobacteria	Gammaproteobacteria	*Enterobacteriales*	*Enterobacteriaceae*	*Klebsiella*	
584a15a8b8273021c7e3e7d07d3bc48d	ILD	Bacteria	Proteobacteria	Gammaproteobacteria	*Betaproteobacteriales*	*Burkholderiaceae*	*Ralstonia*	
7f1495320cf00e9f81e455ea44145d2c	ILD	Bacteria	Proteobacteria	Gammaproteobacteria	*Betaproteobacteriales*	*Burkholderiaceae*	*Achromobacter*	
616cd8134b106d6ed8e9cd6edce9a82a	ILD	Bacteria	Proteobacteria	Gammaproteobacteria	*Pseudomonadales*	*Pseudomonadaceae*	*Pseudomonas*	*Pseudomonas_aeruginosa*
76ba84783044dc63a25709724d12740f	ILD	Bacteria	Firmicutes	Bacilli	*Lactobacillales*	*Streptococcaceae*	*Streptococcus*	
9adcea5ae4bfb4764d7ac5025de92c6d	ILD	Bacteria	Firmicutes	Bacilli	*Lactobacillales*	*Streptococcaceae*	*Streptococcus*	
b15c7ffc9025f0458b58978326cd99b9	ILD	Bacteria	Firmicutes	Bacilli	*Lactobacillales*	*Carnobacteriaceae*	*Granulicatella*	
e7c5690166d106cd3e6f50e7a7f36562	ILD	Bacteria	Firmicutes	Negativicutes	*Selenomonadales*	*Veillonellaceae*	*Veillonella*	
0421e9a57525fc3a89a7c9af9b4c6e4c	ILD	Bacteria	Bacteroidetes	Bacteroidia	*Bacteroidales*	*Porphyromonadaceae*	*Porphyromonas*	
de287dd37785fcd58644cfeeabcd45f4	ILD	Bacteria	Bacteroidetes	Bacteroidia	*Sphingobacteriales*	*Sphingobacteriaceae*	*Sphingobacterium*	*Sphingobacterium_spiritivorum*

(B) Supplementary Figures

Supplementary Fig. S1.Microbiota rarefaction curves showing lung microbiota diversity in ECOPD (red), ILD (green), stable COPD (blue), and sarcoidosis (orange) patient. Each curve shows the average number of species found in a given number of sampled sequences.

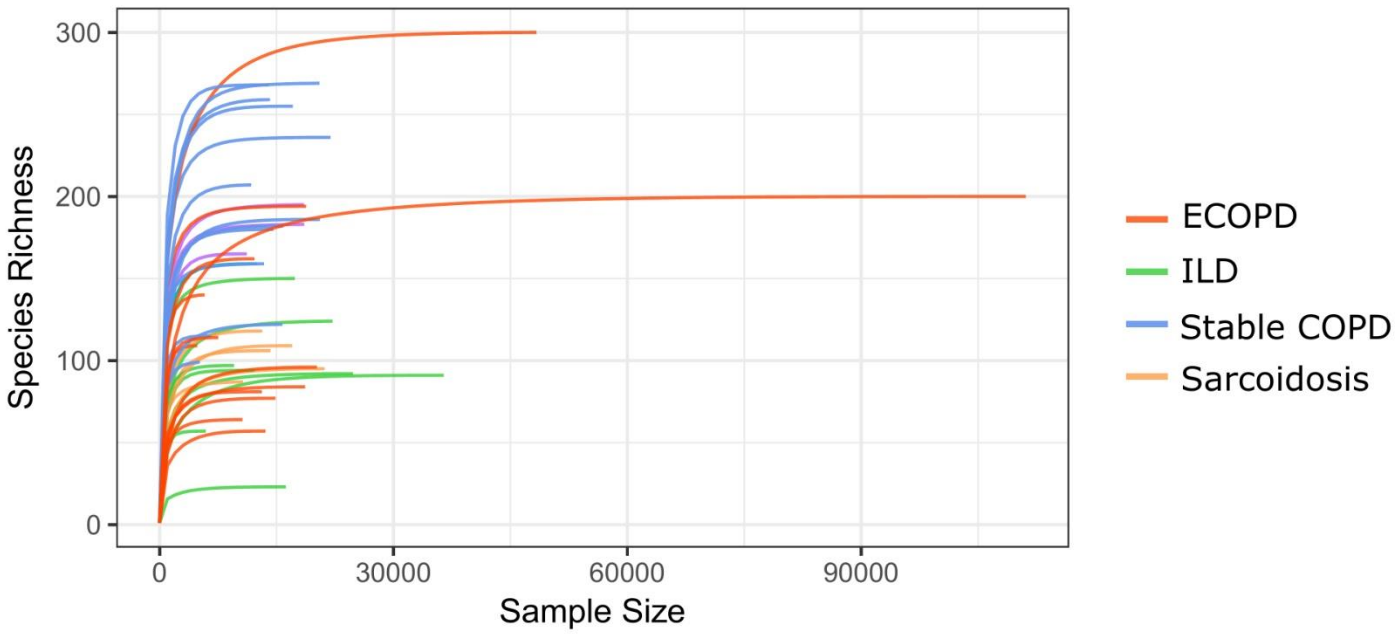

Supplementary Fig. S2. Principal Coordinates Analysis (PcoA) plots of weighted Unifrac distance metrics obtained from sequencing the 16s rRNA gene in lung samples. Axes represent the percentage of data explained by each coordinate dimension.

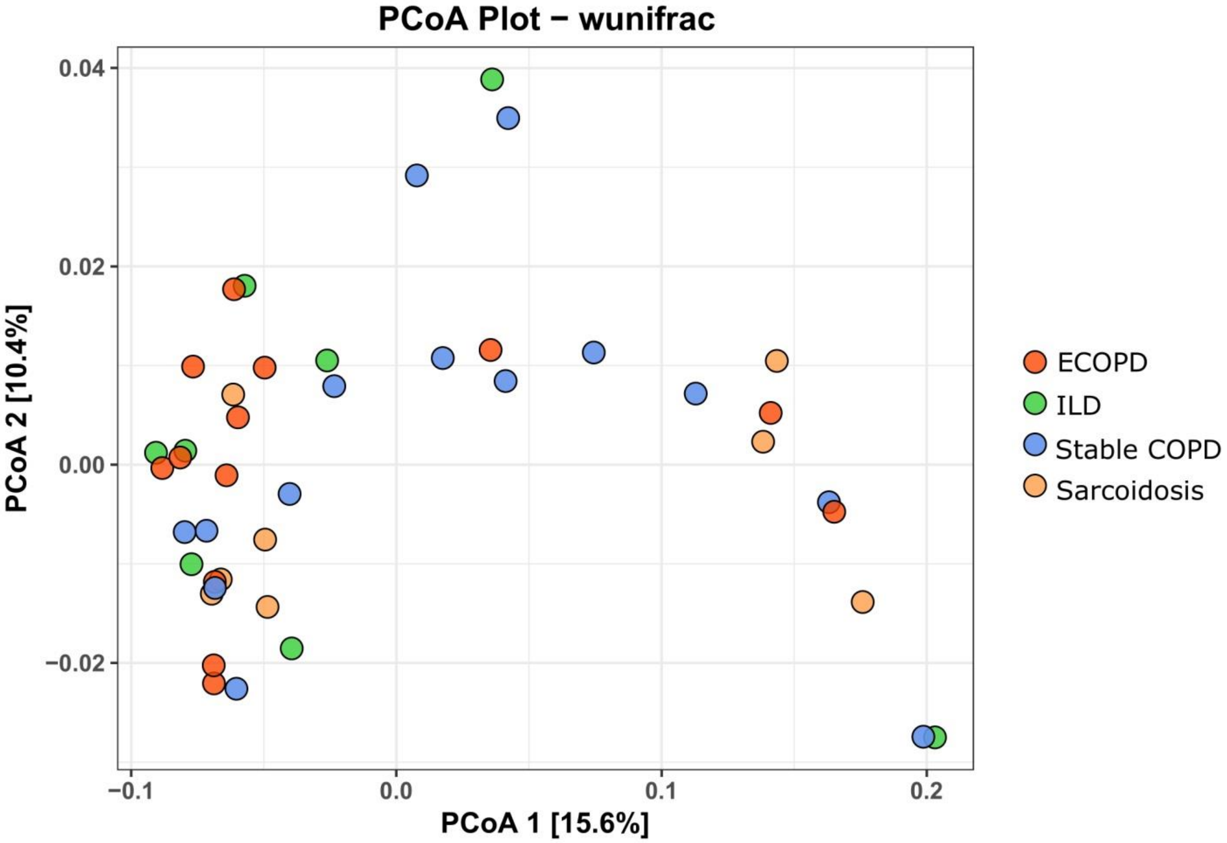

Supplementary Fig. S3. Core lung microbiota of ECOPD, ILD, stable COPD, and sarcoidosis.Venn diagram representation of the ASVs from the different disease groups. The ECOPD, ILD, stable COPD, and sarcoidosis presented 22, 18, 38, and 35 ASVs, respectively. Numbers inside indicate the ASVs shared by two or more groups.

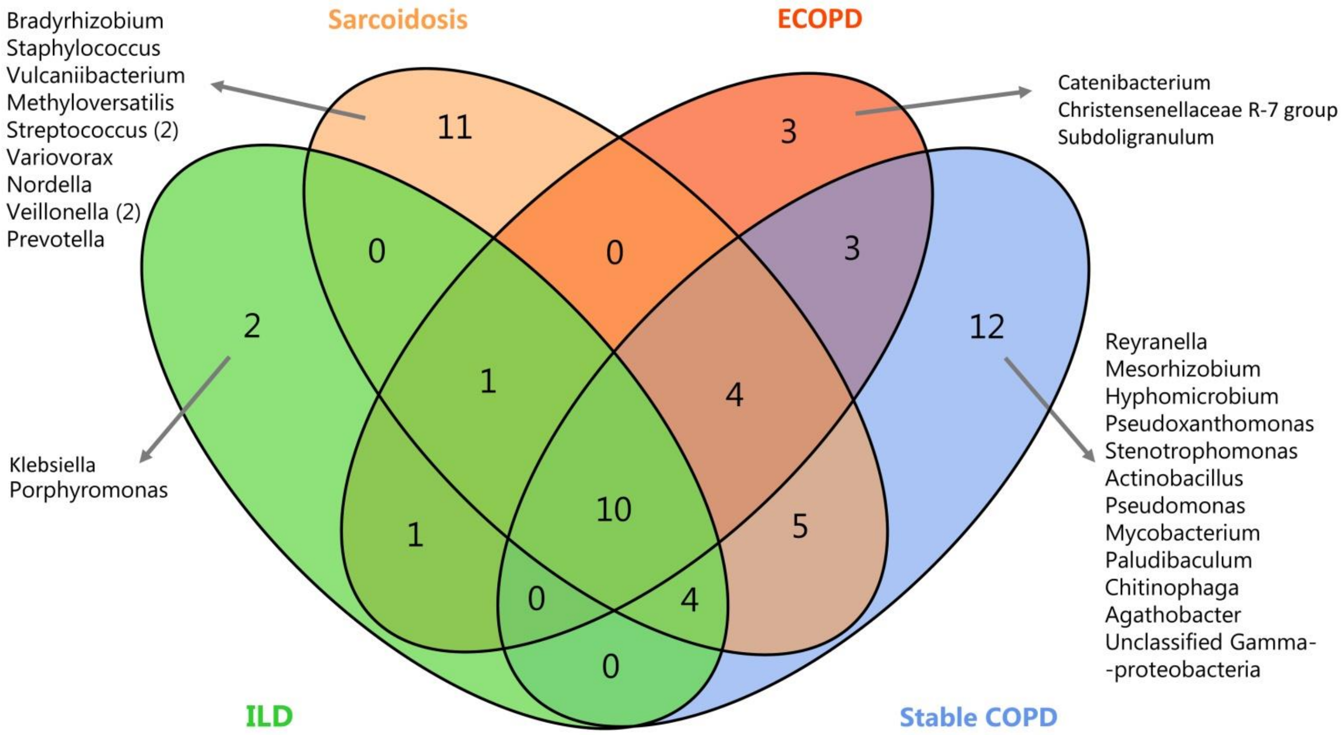

Supplementary Method

Supplementary method SM1. Collection and processing of the negative controls.

Negative control samples (n=3) were collected and processed to check for potential procedural contaminations. Microscopic examination was performed to check for the presence of any microbial contaminants. Additionally, samples were processed in parallel for metagenomic DNA isolation, as well as sequencing library preparation. Neither any microbe nor any DNA was observed in the negative control samples, so samples were not processed further for sequencing. Samples processing reagent control samples (n=2) were processed for sequencing; however no 16S rRNA gene specific sequencing reads were obtained. All samples were processed in a single run to avoid any batch specific sequencing biasness.
